# Triboelectric behaviour of selected zeolitic-imidazolate frameworks: exploring chemical, morphological and topological influences†

**DOI:** 10.1039/d4sc01337a

**Published:** 2024-05-22

**Authors:** Ben Slater, Jin-Chong Tan

**Affiliations:** a Multifunctional Materials & Composites (MMC) Laboratory, Department of Engineering Science, University of Oxford Parks Road Oxford OX1 3PJ UK jin-chong.tan@eng.ox.ac.uk

## Abstract

Tribo- and contact electrification remain poorly understood, baffling and discombobulating scientists for millennia. Despite the technology needed to harvest mechanical energy with triboelectric generators being incredibly rudimentary and the fact that a triboelectric output can be obtained from almost any two material combinations, research into triboelectric generator materials typically focuses on achieving the highest possible output; meanwhile, understanding trends and triboelectric behaviours of related but lower performing materials is often overlooked or not studied. Metal–organic frameworks, a class of typically highly porous and crystalline coordination polymers are excellent media to study to fill this knowledge gap. Their chemistry, topology and morphology can be individually varied while keeping other material properties constant. Here we study 5 closely related zeolitic-imidazolate type metal–organic frameworks for their triboelectric performance and behaviour by contact-separating each one with five counter materials. We elucidate the triboelectric electron transfer behaviour of each material, develop a triboelectric series and characterise the surface potential by Kelvin-probe force microscopy. From our results we draw conclusions on how the chemistry, morphology and topology affect the triboelectric output by testing and characterising our series of frameworks to help better understand triboelectric phenomena.

## Abstract 2

A triboelectric generator harvesting electrical energy from oscillatory mechanical motion. Each grey circle represents a metal–organic framework or counter material used in our study to construct the triboelectric series.

## Graphical abstract

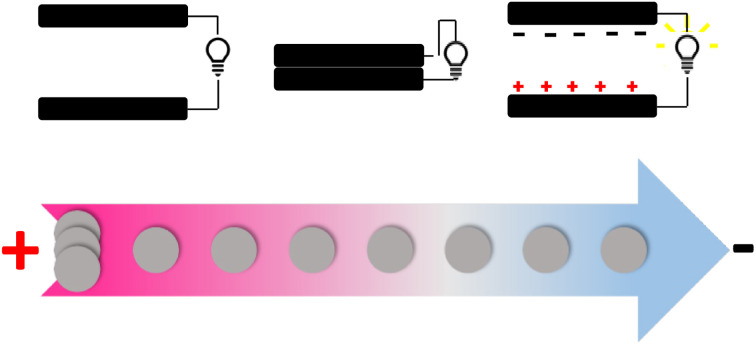

## Introduction

Triboelectric generators represent a class of devices which generate an electrical power output by exploiting the triboelectric effect, whereby frictional or mechanical contact and separation of two or more materials produces an imbalance of charges on the materials. Contact- and triboelectrification has been a long-studied subject; as early as 1926, Shaw reported that like solids can charge each other.^1^ Conversely, triboelectric generators are a relatively new device, first reported by Fan and co-workers in 2012 consisting of a polyethylene terephthalate (PET) and Kapton layer.^2^ Triboelectric generators have their main and most obvious potential application in energy harvesting,^3^ but may also be useful for various other applications in particular self-powered applications such as anticorrosion systems^4^ and human pulse monitoring.^5^ Since the discovery of triboelectric generators, a significant proportion of research in this field has focused on testing various materials for their triboelectric output in triboelectric generators. An excellent strategy is to directly deposit a powdered pure material on conductive tape. In this setup, the powder is directly deposited on the adhesive of the tape and the reverse side (required to be electrically conductive) functions as an electrode. The powder/tape composite can be contact-separated with various counter materials such as PET, PDMS, paper, glass, Kapton or metals to name a few. These counter materials can be easily standardised by using commercially available materials, allowing the community to compare a new material with others previously reported. Typically, the counter material in the device has an electrode attached to the reverse side (referred to herein as the dual-electrode mode).^2,6^ However, this is not strictly required and an alternative measurement method brings the sample in contact with a bare counter material (referred to herein as the single-electrode mode).^7,8^

The powder on a tape setup allows researchers to draw direct conclusions about the triboelectric performance of the material in question. In the literature this strategy has been used for a variety of materials such as flowers,^9^ metal–organic frameworks (MOFs),^10^ milled sunflower husks,^11^ sodium chloride,^12^ and peanut shell powder to name a few.^13^ Similarly, Elvira-Hernández and co-workers effectively created their own copper tape by coating copper with an adhesive glue on which they deposited prickly pear cactus powder.^14^ By another similar strategy, Xie and co-workers deposited instant noodle powder onto double-sided tape which was then stuck to the electrode.^15^ An alternative but arguably more popular strategy involves doping polymer materials with various materials such as cat hair dispersed in poly(vinyl butyrate polymer matrix),^16^ ionic liquid coated single-wall carbon nanotubes embedded in polydimethyl siloxane (PDMS),^17^ clay embedded chitosan films,^18^ polyvinylidine difluoride (PVDF) loaded with ferromagnetic cobalt ferrite,^19^ graphite loaded PDMS,^20^ MOF loaded polymers,^21^ and perfluoro-silane coupling agent modified silica nanoparticles embedded in PVDF.^22^

**Fig. 1:**
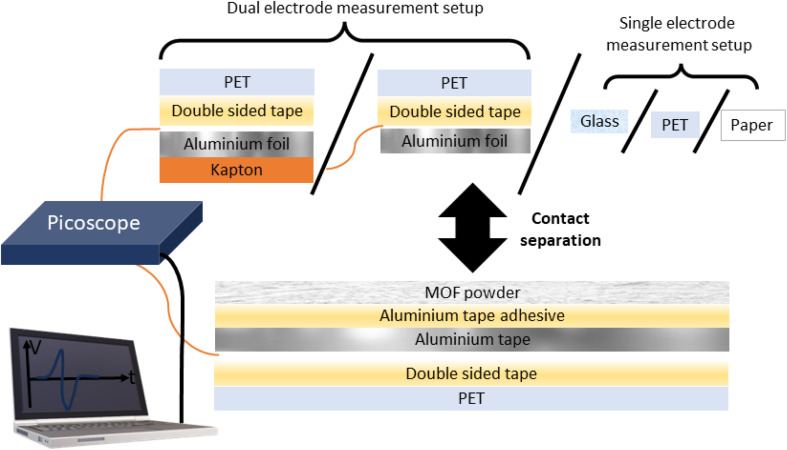
Schematic of the experimental setup used for the triboelectric voltage output measurements, depicting the tape-supported MOF setup and counter material measurement modes for the dual- and single-electrode mode of measurements.

A wide variety of different materials have been tested to date; however, it is often difficult to identify trends and build a fundamental understanding of why materials perform as they do with respect to their triboelectric output. In addition, when a material's triboelectric output is assessed as a polymer matrix, there are likely synergistic and/or knock-on effects between the filler and matrix which may not necessarily originate from the filler material's physical properties. For example, the different phases of PVDF have significantly different electrical activities, and introducing MOFs as fillers was shown to induce the formation of the β phase which has different electrical properties compared to the α phase.^23,24^ We highlight the importance of studying materials in a purer form without a focus on achieving the highest output in order to develop a fundamental understanding of this phenomenon. We propose MOF powders supported on conductive tapes as an exciting material to study for this purpose. MOFs are a diverse group of materials consisting of metal ions or clusters bonded together by organic ligands. Under the MOF umbrella lies zeolitic-imidazolate frameworks (ZIFs), named based on their similarity to aluminosilicate zeolites which have broad structural diversity and excellent stability. We can systematically vary their chemistry, topology, and morphology by consulting the huge library of previously reported methods and procedures. In this work we compare the triboelectric output of 5 ZIF phases supported on conductive aluminium tape, namely, (i) ZIF-8 the sodalite (SOD) topology of Zn(mIm)_2_ [mIm = 2-methylimidazole];^25^ (ii) ZIF-L which is structurally related to the ZIF-8 phase but has 2D layers of the SOD topology and produces ‘leaf’ shaped crystals; in addition ZIF-L has a different chemical formula with some intercalated, protonated 2-methylimidazole, and the reported pre-activation formula is Zn(mIm)_2_·(HmIm)_1/2_·(H_2_O)_3/2_;^26^ (iii) SOD-Zn(CF_3_Im)_2_ [CF_3_Im = 4-(trifluoromethyl)-1*H*-imidazole] exhibits the sodalite topology, isomorphous to ZIF-8 with large sodalite cages;^27^ (iv) quartz (qtz)-Zn(CF_3_Im)_2_, a denser polymorph of the SOD phase;^27^ and (v) ZIF-318, another SOD framework with the formula Zn(mIm)(CF_3_Im) representing a 50% fluorinated framework lying between ZIF-8 and SOD-Zn(CF_3_Im)_2_.^28^ These ZIF-type materials were subjected to contact-separation cycles with various counter materials: aluminium and Kapton (polyimide) in dual electrode mode and glass, PET and paper in single electrode mode (see Fig. 1 for the diagram of the setup). We report the open-circuit voltage (*V*_oc_) and present how these materials fall on a relative triboelectric series to each other and the counter materials. We carefully considered the design and setup of our device and compared them with other powder supported materials in the literature to minimise the interfaces between the electrode and powder which minimises recording any influence of excess interfaces on triboelectric performance. We also considered the setup proposed by Šutka and co-workers who spin coated MOF-solvent suspensions directly onto glass, but due to the long stabilisation times used in our experiments we considered that the weak affinity between MOF powder and glass was not sufficient to last for the high number of cycles used per sample.^29^

In addition, we report the Kelvin-probe force microscopy (KPFM) data for the framework samples, highlighting interesting findings on the surface potential phenomena of these frameworks. Notably, we show how moderation of the CH_3_ to CF_3_ concentration in the three sodalite groups of frameworks can significantly influence the surface potentials and triboelectric nature of the frameworks.

In addition to the interesting triboelectric output findings, we highlight an incidental discovery concerning the mechanochemical synthesis of the Zn(CF_3_Im)_2_ phases. Utilising ball-bearing-vortex grinding and mortar and pestle mechanical grinding apparatus, we show that the previously reported recipe to synthesise the qtz phase,^27^ produces the SOD phase with vortex-ball bearing grinding but the qtz phase when mortar and pestle grinding is used.

## Results

### Synthesis of materials

ZIFs-8, -L and -318 were purchased or synthesised following previously reported methods (as detailed in the Methods section);^28,30^ the powder X-ray diffraction (PXRD) patterns of the formerly mentioned MOFs are all presented in the ESI (see ESI, Fig. S1).† The Zn(CF_3_Im)_2_ phases were synthesised by adapting previously reported procedures from Arhangelskis and co-workers who used a ball milling technique.^27^ The qtz phase was synthesised by ball milling zinc oxide with a protic salt, ammonium nitrate (NH_4_NO_3_). The authors identified challenges to synthesise the SOD phase. Notably, the synthesis of the qtz phase proceeds *via* the SOD phase but the researchers could not reliably control the synthesis and obtain the SOD phase with phase purity. To combat this, they used a more reactive zinc salt, basic zinc carbonate [ZnCO_3_]_2_[Zn(OH)_2_]_3_, without the catalytic salt, NH_4_NO_3_ and obtained the SOD phase with phase purity. A second study in the literature reports the synthesis of these phases also with ball milling. The mechanochemical synthesis of both the qtz and SOD phases was successfully conducted with [ZnCO_3_]_2_[Zn(OH)_2_]_3_; the qtz phase employed methanol as the liquid assisted grinding solvent with NH_4_NO_3_, and the SOD phase employed DMF without the use of NH_4_NO_3_.^31^

In our work, we adapted the procedures from Arhangelskis and co-workers for non-ball milling mechanochemical methods. For the qtz-Zn(CF_3_Im)_2_ synthesis, a solid mixture of reagents was ground in a mortar and pestle for 30 minutes to produce the MOF powder. The first attempt with a feeble hand grinding effort produced a mixed phase containing a small amount of SOD-Zn(CF_3_Im)_2_. Employing a more energetic grinding effort, the qtz phase was obtained without SOD-Zn(CF_3_Im)_2_ impurities (see Fig. S2†). We attempted to adapt this method for vortex grinding using glass vials with ball bearings in a homemade foam holder on a standard lab vortex mixer in a similar setup to that previously reported for MIL 100 (Fe),^32^ and to our surprise this small adaptation produced SOD-Zn(CF_3_Im)_2_ with phase purity (Fig. S3†). We hypothesise that the force experienced during the vortex grinding is much lower than that exerted during hand grinding in a mortar and pestle (and from ball milling as reported in the original method),^27^ which is what dictates the phase formed in our work. We note that the samples used for all experiments herein are the (energetically ground) mortar and pestle synthesised qtz-Zn(CF_3_Im)_2_ and vortex grinder synthesised SOD-Zn(CF_3_Im)_2_. N_2_ adsorption isotherms of SOD-Zn(CF_3_Im)_2_ and qtz-Zn(CF_3_Im)_2_ were recorded at 77 K (see Fig. S4†), and the BET surface areas were calculated to be 457 and 92 m^2^ g^−1^, respectively.

### Triboelectric output

For a control experiment, aluminium foil was used. Aluminium tape supported MOF powder samples were prepared by covering the adhesive layer with MOF powder and removing excess powder by two successive methods according to the Methods section (ESI†). Relative to the other crystal sizes, the synthesis of ZIF-318 produced large rhombic dodecahedral crystals, around 80 microns in length (see the SEM image in Fig. S5†); therefore these crystals were lightly ground in a mortar and pestle prior to deposition on the aluminium tape surface. All other MOF powders were gently manipulated with a spatula to break up any clumps. A summary of selected MOF properties is displayed in Table S1.†

**Fig. 2:**
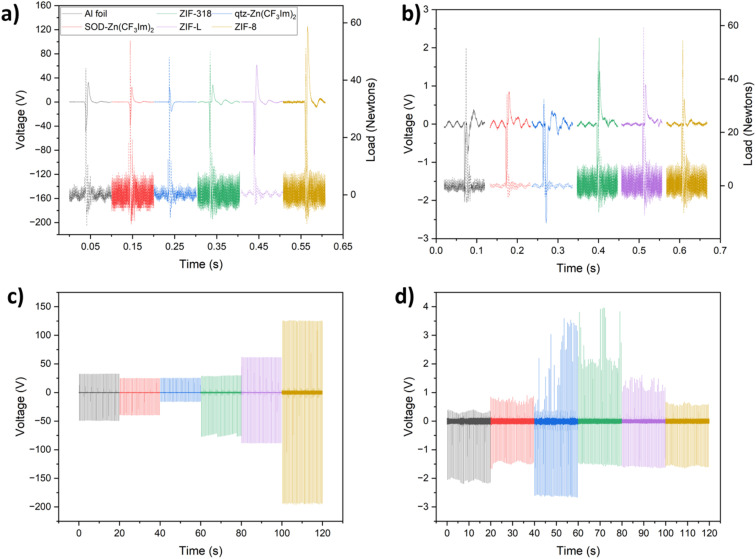
Dual-electrode voltage output of the ZIF series contact-separated against aluminium or Kapton counter materials. Individual peak shapes for all samples are shown for the Kapton counter (a) and aluminium counter (b) materials, and the dashed line represents the force in Newtons as detected by the load cell, plotted on the right *y* axis. The output voltage–time traces are shown for 40 contact-separation cycles against Kapton (c) and aluminium (d) and the colours for each sample are represented by: aluminium foil (black), SOD-Zn(CF_3_Im)_2_ (red), qtz-Zn(CF_3_Im)_2_ (blue), ZIF-318 (green), ZIF-L (purple), and ZIF-8 (yellow).

In addition to analysing the voltage output, we can determine whether the sample gained or lost an electron from/to the aluminium or Kapton counter material by looking at the peak shape, Fig. 2a and b display 100 ms of each voltage–time trace showing a single peak event with the corresponding load cell output; during contact-separation, the load cell measured the force applied (reported in Newtons), and by stacking the triboelectric voltage and load cell outputs we can determine whether there is a positive or negative potential on the sample after separation relative to that of the counter material; this tells us whether the sample tested has lost an electron (positive potential upon separation) or gained an electron (negative potential upon separation) and in turn whether the sample is more tribopositive or more tribonegative than the counter material.

Fig. 2a and c show single peak events and 20 seconds of the voltage-time traces recorded for the series *vs.* Kapton in dual electrode mode. There is a decreasing output trend with increasing fluorination for the 3 SOD phases from ZIF-8 through ZIF-318 to SOD-Zn(CF_3_Im)_2_. This can be attributed to the increasing concentration of CF_3_ groups, increasing the electron accepting ability of the framework, due to its high electronegativity and electron affinity. Conversely, the CH_3_ groups present in ZIF-8 serve as electron donating groups. When comparing the output of SOD-Zn(CF_3_Im)_2_ and qtz-Zn(CF_3_Im)_2_, there appears to be a drop in output when looking at the qtz phase; however, one must not exclusively consider the magnitude of the output, by looking at the peak shape we see that upon separation, SOD-Zn(CF_3_Im)_2_ and all other MOFs except for qtz-Zn(CF_3_Im)_2_ have a positive separation peak corresponding to a loss of electrons from the MOF sample. However, qtz-Zn(CF_3_Im)_2_ has a negative separation peak meaning that this sample gained an electron from the Kapton counter material. Originally what appeared as a decreased output was almost overlooked, but we were surprised to find that there was a reversal in the triboelectric behaviour, which we attributed to the higher fluorine density with the change in topology from SOD to qtz. We observed a lower triboelectric output for ZIF-L compared with ZIF-8. This indicates that there are possible morphological influences on the triboelectric behaviour. However, it is important to note that ZIF-L has a different chemical formula and water content compared to ZIF-8 which also influences the hydrophilicity of the material.

Fig. 2b and d show the corresponding voltage output and peak shape for the MOFs *vs.* aluminium counter material in dual-electrode mode. There is a much lower voltage output for these measurements, and we are unable to draw any conclusions from comparing the outputs of the different MOFs due to variation in voltage output for the same sample. Nevertheless, the electron transfer behaviour could still be studied by analysing the separation-peak shapes. As reported for the measurements with the Kapton counter material, all samples have a positive separation peak except for qtz-Zn(CF_3_Im)_2_ which has a negative separation peak. We also note that there is a ‘pseudo’ voltage output picked up by the oscilloscope, a consequence of the electromagnetic shaker used to contact-separate the two materials. This is noticeable for the aluminium counter low voltage measurements; for example, the SOD-Zn(CF_3_Im)_2_ sample in Fig. 2b has a negative output voltage which starts before the load cell registers force. This was confirmed by operating the magnetic shaker without bringing the sample in contact with the counter material and observing a voltage output as shown in Fig. S6 and S7.† However, importantly this interference happens before the separation peak, and hence it does not influence it but it is important to note it for clarity.

**Fig. 3:**
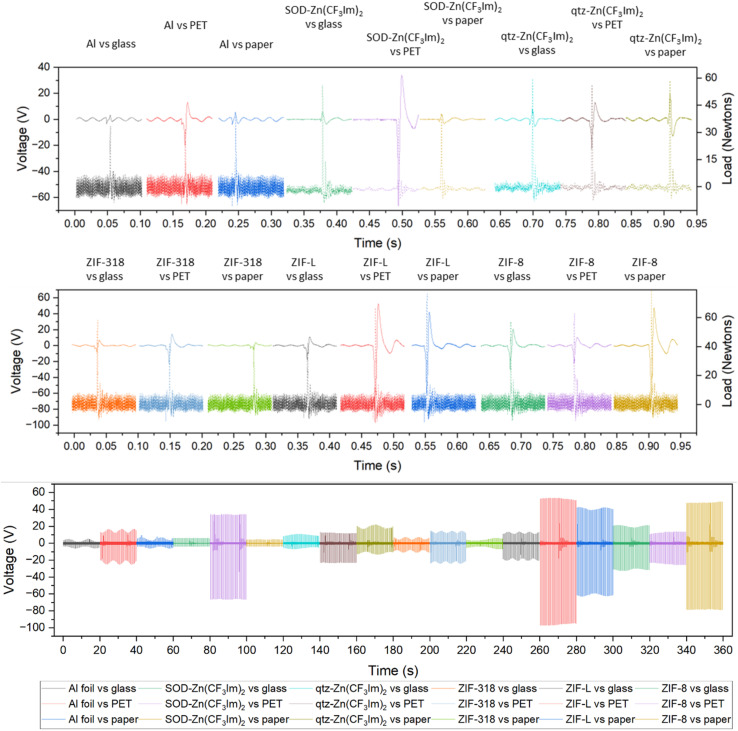
Single-electrode voltage output of the ZIF series against three counter materials: glass, paper and PET. Peak shapes for single peaks are shown in the top and middle plots, where the dashed line represents the force as detected by the load cell, plotted on the secondary *y* axis. Voltage–time traces in the bottom plot are shown for 40 contact-separation cycles for aluminium foil *vs.*: glass (black), PET (red), paper (blue); SOD-Zn (CF_3_Im)_2_*vs.*: glass (green), PET (purple), paper (yellow); qtz-Zn(CF_3_Im)_2_*vs.*: glass (turquoise), PET (brown), paper (olive); ZIF-318 *vs.*: glass (orange), PET (blue), paper (green); ZIF-L *vs.*: glass (black), PET (red), paper (blue); and ZIF-8 *vs.*: glass (green), PET (purple), paper (yellow).

To better understand the triboelectric behaviour, we also measured the triboelectric voltage output of aluminium foil and the five MOFs against three other materials in single electrode measurement mode to compare the MOFs with benchmark materials and create a triboelectric series; these data are displayed in Fig. 3. Each sample was contact-separated with glass, PET and paper, and the voltage output was recorded from an electrode attached to the reverse of the sample as illustrated in Fig. 1. The triboelectric voltage output was cross-referenced with the load cell output as described for the dual-electrode measurements. For all measurements *vs.* PET, a positive potential was observed on the sample as referenced by the positive peak shape upon separation. This indicates that PET withdraws electrons from all samples, and hence PET is the most tribonegative material relative to all other materials. SOD- and qtz-Zn(CF_3_Im)_2_ both displayed negative separation peaks against the paper and glass counter materials indicating that these MOFs are more tribonegative and withdrew electrons from paper and glass. The paper and glass counter materials both showed the same triboelectric behaviour towards ZIF-318, ZIF-L and ZIF-8, indicating that the MOFs are electron donors and tribopositive relative to paper and glass. There were variations in the magnitude of the voltage output of these three frameworks *vs.* the individual counter materials, but we were unable to identify any voltage output trends. To further differentiate the behaviour of paper and glass with respect to each other, a paper sample was prepared (electrode on the reverse of paper) and tested against PET and glass, and these results are displayed in Fig. S8† (full voltage-time traces) and Fig. S9† (peak shape with the load cell output) showing that paper donates electrons to PET and withdraws electrons from glass. There were two combinations where it was not possible to identify a separation peak in the voltage–time trace, these were aluminium foil *vs.* glass, and aluminium foil *vs.* paper where no voltage peaks were recorded upon separation.

**Fig. 4:**
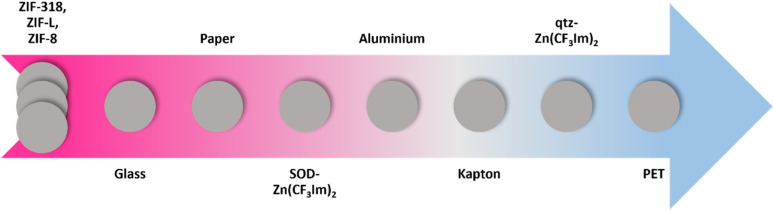
The triboelectric series determined from this work. The series goes from the most tribopositive (electron donating) in pink through grey to the most tribonegative (blue). ZIF-8, ZIF-L and ZIF-318 represent MOFs which could not be quantified in the series with respect to each other due to their similar triboelectric behaviours as detailed in the discussion.

From the dual- and single-electrode voltage output peak shape analyses, we were able to place the MOFs and counter materials on a relative triboelectric series as shown in Fig. 4. For all material combinations tested, the material further to the right withdraws electrons from the material on its left in the series. This was true for all combinations except for the two previously discussed scenarios (aluminium *vs.* glass and aluminium *vs.* paper).

We strongly emphasise that this triboelectric series has been formulated based on the electron loss/gain phenomena only and not the magnitude of the triboelectric voltage output. Quantification in the series was purely deduced from the triboelectric behaviour against the counter materials used in this study. In most cases, material-counter sample pairs further away from each other in the series produce higher outputs, but some pair outputs do not follow the series. For example, ZIF-8 did not have the highest performance against PET in single-electrode mode which was expected from the series; this material pair combination had an unexpectedly low output. Although there are apparent differences in the properties of the MOFs, aside from the previously discussed effect of the fluorine concentration in the 3 SOD phases increasing the electron accepting ability, the changes in triboelectric output and behaviour were difficult to associate with specific framework features, and therefore we measured the relative surface potential by Kelvin probe microscopy to probe the surface material's properties at the nanoscale.

### Kelvin-probe force microscopy of MOF pellets

The relative surface potential of the 5 MOF frameworks was characterised by KPFM; aluminium was also scanned as part of this work and literature values for Kapton and PET were used for reference. The MOF powders were pressed into 13 mm pellets using a die press; we chose to measure the materials as pellets so that they would first, provide a flattish surface for scanning probe microscopy and second, produce a densely populated surface *via* a solvent-free processing method with a range of orientations of the powders (although this was not achieved for ZIF-L for reasons explained below). To form the pellets, a force of 7 tonnes was applied corresponding to a pressure of 520 MPa for 10 minutes. PXRD patterns were recorded for the pressed pellets to ensure that the MOF powders remained crystalline; these patterns are shown in Fig. S1.† As expected, due to mechanical deformation there were differences in the pellet PXRD patterns compared with those of the as-synthesised materials for the different frameworks. For all materials, there were losses in crystallinity of varying degrees, as referenced by the broadening of peaks in the pelletised sample PXRD patterns and decrease in the signal to noise ratio compared with those of the as-synthesised sample. The PXRD patterns of the pelletised samples were recorded in reflection mode with an incident angle of 0° being perpendicular to the uniaxial force of pelletisation. Interestingly, the ZIF-L particles became mostly ordered during pelletisation; this is apparent crystallographically from the increase in relative intensity of the peak at 2*θ* = 7.4° corresponding to the *h*, *k*, *l* plane of (200), which is located on the leaf shaped particle face. Conversely, the peak at 2*θ* = 8.9° corresponding to the (002) plane was completely absent in the pelletised MOF pattern, and the (002) plane is perpendicular to the leaf surface which is why it is supressed in the PXRD pattern of the pellet. Furthermore, the topography (recorded with KPFM) shows the scanned area of the face in parallel to the uniaxial pelletisation force. Comparing the scanning electron micrograph of the bulk powder of ZIF-L (Fig. S10†) with the amplitude-modulated mode atomic force micrograph of the ZIF-L pelletised sample (Fig. S11†), there is a clear orientation preference of the ZIF-L particles to be leaf face up upon pelletisation. For KPFM scans, all pellets were mounted with a solid setting wax. We stress that these are not quantitative methods as no calibration was conducted with a known standard. However, because all samples were measured with the same tip and measurement conditions, they can be compared relatively.

**Fig. 5:**
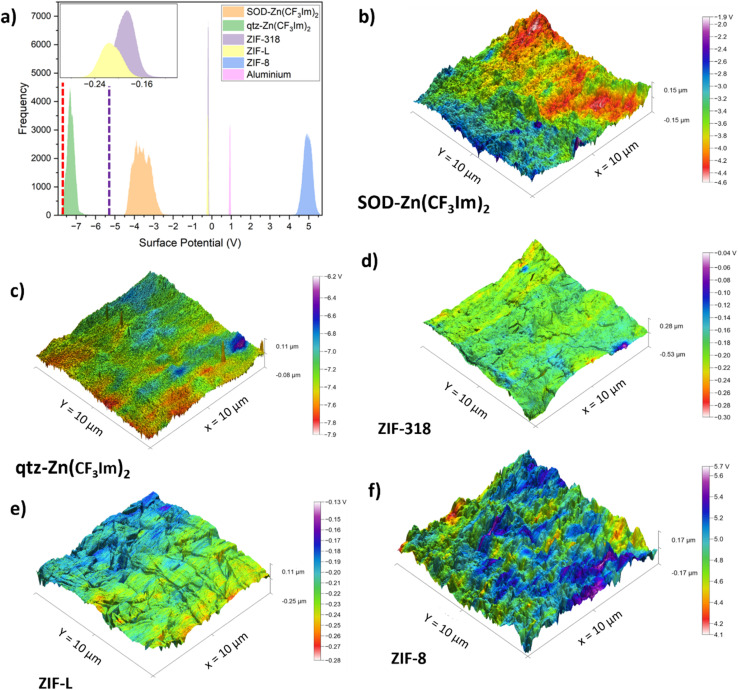
Kelvin-probe force microscopy data displayed as (a) histograms of each KPFM image of the MOF series and aluminium tape (conductive side), literature values for the average KPFM measured surface potentials of PET and Kapton are shown as dashed lines (red and purple respectively), and the inset displays a focused view of the ZIF-318 and ZIF-L histograms. (b–f) 3D maps of the height profile topography with the KPFM surface potential overlaid as pseudocolour with the corresponding voltage-colour scales.

The histograms shown in Fig. 5a show that the surface potential correlates well with the triboelectric series; the MOFs follow the trend of the triboelectric series with ZIF-8 and qtz-Zn(CF_3_Im)_2_ at opposite ends of the surface potential histogram. Literature values for KPFM surface potential measurements of PET-7.6 V,^33^ and Kapton-5.415 V,^34^ also conform to our triboelectric series trend and are shown as dashed lines in Fig. 5a (red and purple respectively). The KPFM surface potential of the aluminium sample measured as part of this work, is the only sample which doesn't correlate with the triboelectric series trend; it has a more positive surface potential than SOD-Zn(CF_3_Im)_2_, ZIF-L and ZIF-318, yet aluminium falls to their the right on the triboelectric series. Reasons for the discrepancies may be due to the different states (continuous foil *vs.* pelletised powder) and the fact that aluminium is an electrical conductor. The 3D height profile with KPFM output overlaid for the aluminium sample is displayed in Fig. S12.†

Whilst the samples reported here were not contact-electrified prior to KPFM measurements, we noticed similar charge distribution patterns to those described by Baytekin and co-workers who reported a mosaic of surface charges.^35^Fig. 5b–f show the 3D height sensor maps with the KPFM output overlaid, and the charge distribution appears to be random with mosaic type patterns. We also note that surface features such as topographic changes shown in the 3D height sensor micrographs cannot be correlated with surface potential ‘hotspots’ or ‘coolspots’ of high/low surface potential. Furthermore, the hot/coolspots are collections of many randomly oriented crystals and cannot be correlated with individual crystals or specific crystal orientations. Fig. S13† shows the roughness parameter of each MOF pellet sample plotted against the surface potential of each sample. This shows that, first, the roughness of the MOF powders is of the same order of magnitude and second, that there is no correlation between surface potential, triboelectric behaviour and surface roughness. Fig. S11 and S14–S18† show the corresponding amplitude modulated mode AFM images to highlight representative surface morphologies evident on the pelletised samples. We also report KPFM scans of the single crystals of ZIF-318 and discuss this in the ESI.†

## Conclusions

In summary, we have successfully adapted the synthesis of two Zn(CF_3_Im)_2_ phases to rudimentary mechanochemical synthesis techniques and made an incidental discovery concerning the effect of the force used during the synthesis, which dictates whether the phase obtained with the previously reported qtz-Zn(CF_3_Im)_2_ recipe yields the qtz phase (forceful mortar and pestle grinding) or SOD phase (vortex mixer grinding). We assessed the triboelectric behaviour of five selected ZIF phases supported on aluminium tape and were able to place these materials in a triboelectric series with other typical triboelectric materials based on the triboelectric behaviour alone. Furthermore, we correlated the triboelectric behaviour with surface potential data recorded by KPFM. The most significant effect on the triboelectric behaviour and surface potential was –CH_3_ to –CF_3_ functional group modification of the three SOD frameworks. ZIF-318 and SOD-Zn(CF_3_Im)_2_ became increasingly tribonegative and there are drastic changes in the surface potential of these MOFs from positive to negative. Further topological influences were investigated by comparing two topologically related pairs of frameworks: ZIF-L and ZIF-8, and the qtz and SOD phases of Zn(CF_3_Im)_2_. ZIF-L is topologically related to ZIF-8 but has a 2D layered structure and morphologically unique leaf-shaped particles; this caused large changes in the triboelectric behaviour and surface potential as discussed. qtz-Zn(CF_3_Im)_2_ has a denser structure which further contributes to a higher fluorine density in the framework, meaning that the qtz framework has quite different triboelectric behaviours and a more negative surface potential. In addition, KPFM scanning of ZIF-318 single crystal facets showed little variation in surface potential of the {110} family of *h*, *k*, *l* planes where a total of at least 3 different crystal facets were scanned. We hope that this research inspires others to investigate the triboelectric behaviour more carefully when reporting their research instead of just considering improvements in the triboelectric output. We call attention to the information which can be obtained from the separation peak in particular and hope that others will adopt this method in the future.

## Author contributions

BDS: conceptualisation, methodology, investigation, writing – original draft, and visualisation. JCT: writing – review and editing, supervision, project administration and funding acquisition.

## Conflicts of interest

There are no conflicts to declare.

## Supplementary Material

SC-015-D4SC01337A-s001
