# Supplementary material for: Triboelectric behaviour of selected zeolitic-imidazolate frameworks: exploring chemical, morphological and topological influences
Source: Chem Sci. 2024 May 22;15(26):10056–64. doi: 10.1039/d4sc01337a (PMC11220587; doi:10.1039/d4sc01337a)
Supplement: SC-015-D4SC01337A-s001 [file SC-015-D4SC01337A-s001.pdf]

Electronic Supplementary Material (ESI) for Chemical Science.
This journal is © The Royal Society of Chemistry 2024

Supplementary Information

Triboelectric Behaviour of Selected Zeolitic-Imidazolate Frameworks Exploring Chemical, Morphological and Topological Influences

Ben Slater and Jin-Chong Tan

Multifunctional Materials & Composites (MMC) Laboratory, Department of Engineering Science,

University of Oxford, Parks Road, Oxford OX1 3PJ, United Kingdom.

Materials

For dual-electrode contact separation mode, counter materials were prepared as follows and according to Figure 1: aluminium foil was cut into 2.5 cm x 3 cm, double-sided tape stuck the aluminium foil to a PET supporting piece. A wire was fixed between the aluminium and double-sided tape which was attached to the oscilloscope for measurements. For the Kapton counter, an electrode was prepared as described above and then a 2.5 cm x 3 cm piece of Kapton tape was used to cover the aluminium.

For single-electrode measurements, PET sheets were obtained from Lloyd Platon overhead projector A4 photocopier film with a measured thickness of 100 µm. And cut into 3 cm x 3.5 cm pieces. The reverse side was supported by double-sided tape. Paper was cut from MyCopy Professional A4 White Copier Paper into 3 cm x 3.5 cm pieces. The reverse side of the paper was supported by double-sided tape for attachment. The glass used was a Fisher model 1238-3118 glass microscope slide with a reported thickness of 1.0-1.2 mm. The full slide was used for the single electrode measurements supported by double-sided tape on the reverse side.

MOF sample electrodes were prepared as follows; for both single- and dual- electrode mode: aluminium tape (19 mm width) was cut into 20 mm lengths to give an active area of 380 mm^2. The foil side was stuck to a PET support with double sided tape and a wire was attached between the foil and double-sided tape. The MOF powder was spread on the aluminium tape adhesive side, it was ensured that all adhesive was covered by spreading the MOF powder well. After which, the excess was removed by a handheld gas blower (camera cleaner bulb type) and then Sellotape was used to lift excess powder. The tape adhesive was fully covered by a layer of MOF, the mass difference before and after loading was below 1 mg and was not quantified. The aluminium sample reported was prepared from 1.9 cm x 2 cm aluminium foil and supported by a PET sheet and double-sided tape.

ZIF-8 was purchased from BASF (Basolite Z1200). The PXRD pattern was recorded before measurements to ensure crystallinity of the material after storage (Figure S1). All other chemicals were purchased from commercial suppliers and used as supplied without purification.

Methods

Synthesis of SOD-$Zn(CF_3Im)_2$ by vortex grinding

Reagents and quantities as reported by Arhangelskis and co-workers for the ball milling synthesis of qtz- $Zn(CF_3Im)_2$ were used.[1] Briefly, ZnO (40.69 mg, 0.5 mmol), HCF_3Im (138.8 mg, 1.0 mmol) and catalytic salt ammonium nitrate (NH_4NO_3, 10 mg, 0.125 mmol) were placed in a 20 ml glass vial (27 mm diameter), to this solid mixture, 50 µl of DMF and three zirconia ball bearings (4 mm diameter) were added and the mixture was vortexed at 1200 rpm for 30 minutes in a homemade foam holder

attached to the vortex grinder (see photo in Figure S19). N.B. two additional separate syntheses were conducted using the same conditions as above except:

1) Stainless steel ball bearings replace zirconia

2) the same reagents as above but without catalytic NH_4NO_3

Synthesis of qtz-Zn(CF_3Im)$_2$ by hand grinding

Reagents and quantities as reported by Arhangelskis and co-workers for the ball milling synthesis of qtz-Zn(CF_3Im)$_2$ were used.[1] Briefly, ZnO (40.69 mg, 0.5 mmol), HCF_3Im (138.8 mg, 1.0 mmol) and catalytic salt NH_4NO_3 (10 mg, 0.125 mmol) were placed in a 95 mm internal diameter agate mortar and pestle. 50 µl of DMF was added and the mixture was hand ground for 30 minutes. (N.B. a forceful grinding force was used which caused mild hand blisters).

Post-treatment of SOD- and qtz-Zn(CF_3Im)$_2$

The synthesised powders were centrifuged three times T 10000 RPM for 10 minutes with fresh methanol and dried under vacuum at room temperature overnight.

Synthesis of ZIF-318

ZIF-318 was synthesised following the previously reported synthesis method from Mondal and co-workers with minor adaptions.[2] Reagents and solvent were doubled and zinc nitrate hexahydrate was used in place of the tetrahydrate salt. 0.44 mmol of 2-methylimidazole (36 mg), 0.44 mmol of 2-trifluoromethylimidazole (59.8 mg) and 0.44 mmol of zinc nitrate hexahydrate (130.9 mg) were dissolved in 6 ml of diethylformamide and the mixture was sealed in a 20 ml Teflon lined stainless steel reactor and added to an oven at 135°C for 48 hours, after which the oven was switched off and the reactor was cooled naturally in the oven. The product was obtained and washed with diethylformamide and ethanol, respectively. The crystals were air dried.

Synthesis of ZIF-L

ZIF-L was synthesised by following the previously reported procedure from Low and co-workers.[3] 1.97 mmol or zinc nitrate hexahydrate (0.586 g) and 15.8 mmol of 2-methylimidazole (1.298 g) were each separately dissolved in 40 ml of deionised water. Then, the zinc solution was added to the 2-methylimidazole solution under stirring on a hot plate set at 30°C. The crystals were centrifuged at 10,000 rpm and washed twice with deionised water and twice with methanol (with centrifugation between each step) before being dried under vacuum at 50°C.

Preparation of MOF pellets and KPFM samples

A-13 mm die press (Specac) was used without employing the vacuum feature to prepare the pellets. 2-3 small spatulas of MOF powder were added into the die press and a weight of 7 tons was applied for 10 minutes, equating to 520 MPa. A piece of each pelletised MOF or single crystals of ZIF-318 were mounted to the AFM metallic stubs with mounting wax (Agar Scientific 80°C melting point). The mounting wax was melted on the stub at 100°C before the MOF pellet piece a small amount of ZIF-318 single crystals were added. The stub was then quickly removed and cooled to set the wax. Samples were not ground.

The aluminium sample was prepared by sticking aluminium tape directly on the KPFM metal stub with ground wire. Silver tape was used to make an electrical connection between the conductive tape surface and metallic stub. The ground wire was ground to the recommended ground connection of the Cypher AFM.

Powder X-ray diffraction (PXRD)

PXRD patterns were recorded on a Rigaku Miniflex powder diffractometer with Cu Kα radiation. All patterns were normalised and compared with the normalised, simulated patterns from the corresponding CIF files obtained from the Cambridge Structural Database (CSD), the CSD references to the corresponding CIF files are given in

Table S1. Varying scan speeds were used but a step size of 2θ = 0.01° was used for all measurements. All crystals except for ZIF-318 crystals were analysed as-synthesised after drying. ZIF-318 crystals were ground in a mortar and pestle prior to measurements. Pelletised samples were measured by placing a piece of pelletised MOF on the glass PXRD slide.

Scanning electron microscopy (SEM)

SEM micrographs were recorded on a Hitachi TM3030Plus Tabletop Microscope with 15 kV acceleration voltage under vacuum. The samples were mounted onto carbon tape supported on aluminium stubs.

Triboelectric measurements

The open circuit voltage measurements were recorded on a Picoscope 5000 series. For dual-electrode measurements the sample's electrode was connected to the oscilloscope probe tip and the counter material electrode was connected to the probe's ground. For single-electrode measurements, the oscilloscope probe tip was connected to the sample's electrode whilst the ground tip was connected to ground. A Ling Dynamic Systems (LDS) model V201 electromagnetic shaker was used to apply force for the contact-separation measurements. The frequency and duty (percentage of time the materials were in contact relative to the time of a cycle) were set by a Gwinstek AFG 2105 arbitrary function generator. For all experiments, a frequency of 2 Hz and a duty of 2% were used. A Thandar T32021S power supply was set to 23 V to power the magnetic shaker. The force was measured by a RS Pro stainless steel S beam tension cell (500 kg range) connected to a DC power supply PS-304 II. The output was monitored by the Picoscope 5000 series oscilloscope. The load cell output was calibrated and the corresponding calibration curve is shown in Figure S20. N.B. some load measurements had a 1000 Hz noise filter activated and some did not, this is apparent when comparing the signal to noise ratio of different traces, nevertheless, all data has a sufficient signal to noise ratio. Before data reported here was measured, the triboelectric output voltage was stabilised by performing repeat contact separations at 2 Hz for at least 30 minutes.

Kelvin probe force microscopy (KPFM)

KPFM scans and corresponding measurements were recorded on an Asylum Research Cypher ES atomic force microscope (AFM), with an Asylum Research ASYELEC-01-R2 conductive tip. The scan conditions for the pelletised samples were as follows: 1024 line scans were performed at 0.5 Hz in two-pass mode, at a delta height of 25 nm. The single crystals scans of ZIF-318 were recorded with the same tip and scan conditions as described above, but only 512 line scans were performed.

N.B. The scans published in this work were all recorded after stabilisation where we would record several scans prior to the reported scan at a lower resolution, typically 128 line scans. This is due to the first scans having a significant drift in surface potential from the start to the end of the scan. Typically at least 4 scans were recorded prior to measurement and we would ensure that there was no noticeable drift in the scan from start to finish before measuring the high resolution scans reported here.

The surface roughness was calculated using Gwyddion software statistical analysis moment based feature. The root mean square was used to determine each material's roughness.

Gas sorption and BET surface area determination

Brunauer-Emmett-Teller (BET) nitrogen adsorption isotherms were recorded at 77 K using a Quantachrome Autosorb iQ instrument, 60 to 100 mg of sample was used and samples were activated at 150°C under vacuum prior to analysis.

Surface accessible volume (SAV) calculations

The solvent accessible surface was calculated using CSD Mercury software's Voids feature. The probe radius was set to 1.2 Å and the grid spacing to 0.3 Å.

Supplementary discussion on the addition of NH_4NO_3 and synthesis with stainless steel ball bearings

Synthesis without catalytic salt NH_4NO_3 produced a product with higher zinc oxide impurity as shown in Figure S3. The purple pattern represents the sample prepared by vortex grinding without addition of NH_4NO_3 and shows prominent peaks corresponding to zinc oxide at 2θ = 32°,34.5° and 36.3°. The samples prepared with zirconia (green pattern) and stainless steel (yellow pattern) ball bearings matched the simulated PXRD pattern of SOD-Zn(CF_3Im)$_2$. There were also trace impurities of zinc oxide in these two PXRD patterns, zinc oxide is insoluble in water and organic solvents and cannot be removed by washing steps. The ratio of the SOD peak height at 2θ = 7.2° to the most prominent zinc oxide peak at 2θ = 36.3° were 1:0.16, 1:0.056 and 1:0.035 for the zirconia without NH_4NO_3 zirconia with NH_4NO_3 and stainless steel with NH_4NO_3 respectively. We chose to use the zirconia ball bearings as the stainless-steel ones corroded. Zinc oxide impurities are also visible in the PXRD patterns using ZnO as starting material published by Arhangelskis and co-workers.[1]

Supplementary discussion single-crystal ZIF-318 KPFM

In addition to the pelletised samples, we present two KPFM scans of single crystals of ZIF-318. Crystals of around 100 µm were directly mounted on solid setting wax and the KPFM scans were recorded. We did not determine the *h,k,l* planes that were being scanned, however, we scanned a crystal edge with 2 corresponding *h,k,l* planes as shown in Figure S21 and a vertex with the 3 corresponding *h,k,l* planes as shown in Figure S22. These facets all belong to the (110) family and are expected to be crystallographically identical but likely to differ in local defects. Parts of both images suffer from scanning artefacts caused by large changes in height in the crystal. Nevertheless, there are portions of each 2 or 3 faces which scanned satisfactorily and we can conclude that at least three *h,k,l* planes have been scanned. No notable differences in surface potential of the scanned planes were observed.

Table S1 Samples tested for their triboelectric output in this work.

**Sample**	**2-methylimidazole equivalence**	**2-trifluoromethylimidazolate**	**Topology**	**Density***	**Synthesis method**	**Solvent accessible surface (1.2 Å probe radius)**	**CIF Reference (CSD)**
Aluminium foil	-	-	-	2.7 (nominal value for pure Al(	-	-	-
ZIF-8	1	0	Sodalite (SOD).	1.17	BASF proprietary information	25.7%	739161
ZIF-L (leaf)	1	0	2D layers of SOD topology.	1.45	RT liquid	0%	1509273
ZIF-318	0.5	0.5	SOD	1.52	Solvothermal	21.9%	1495488
SOD-$Zn(CF_3Im)_2$	0	1	SOD	1.24	Vortex grinding	22.3%	1859152
Qtz-$Zn(CF_3Im)_2$	0	1	Quartz (qtz)	1.95	Hand grinding	0%	1859151

*Density of MOFs reported with CIF files

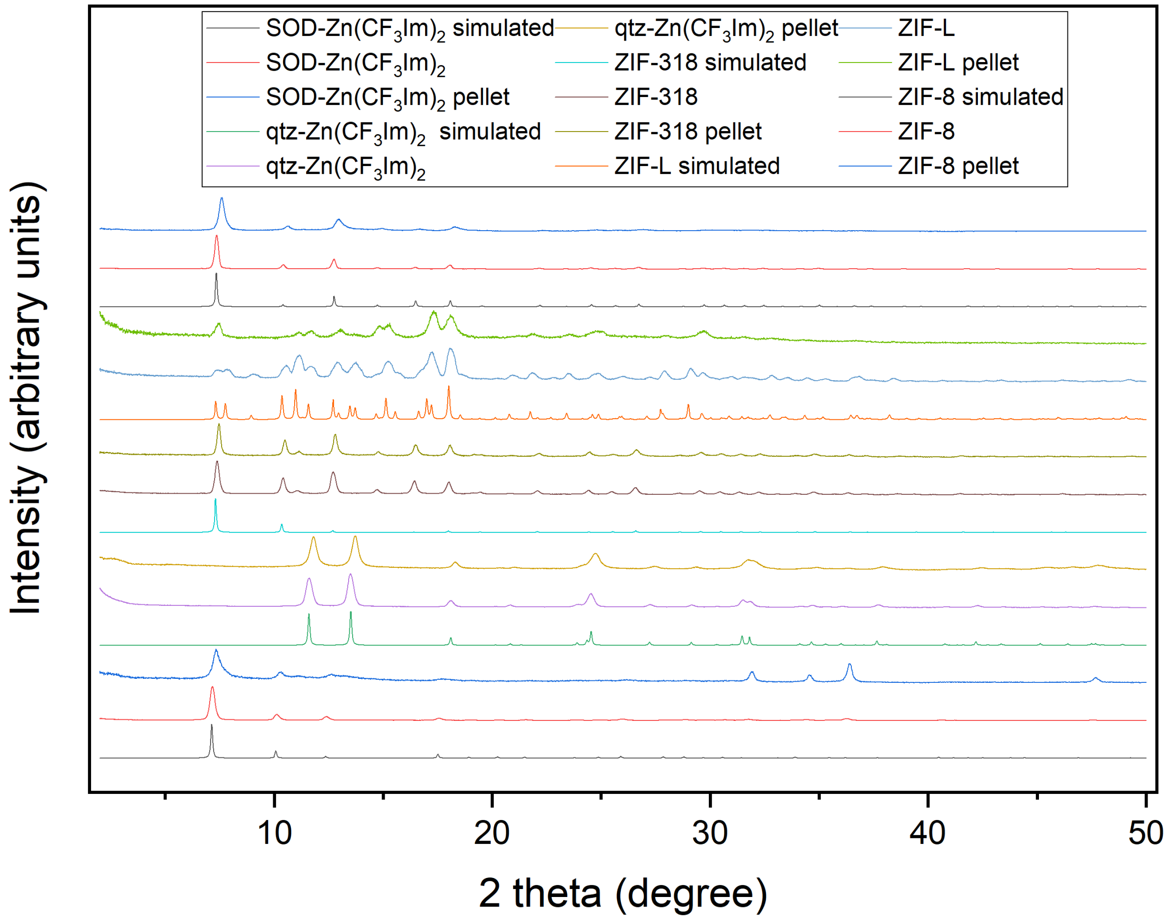

Figure S1. PXRD patterns of the: simulated patterns, as-synthesised/purchased samples and the pelletised samples of the 5 ZIFs used in this work.

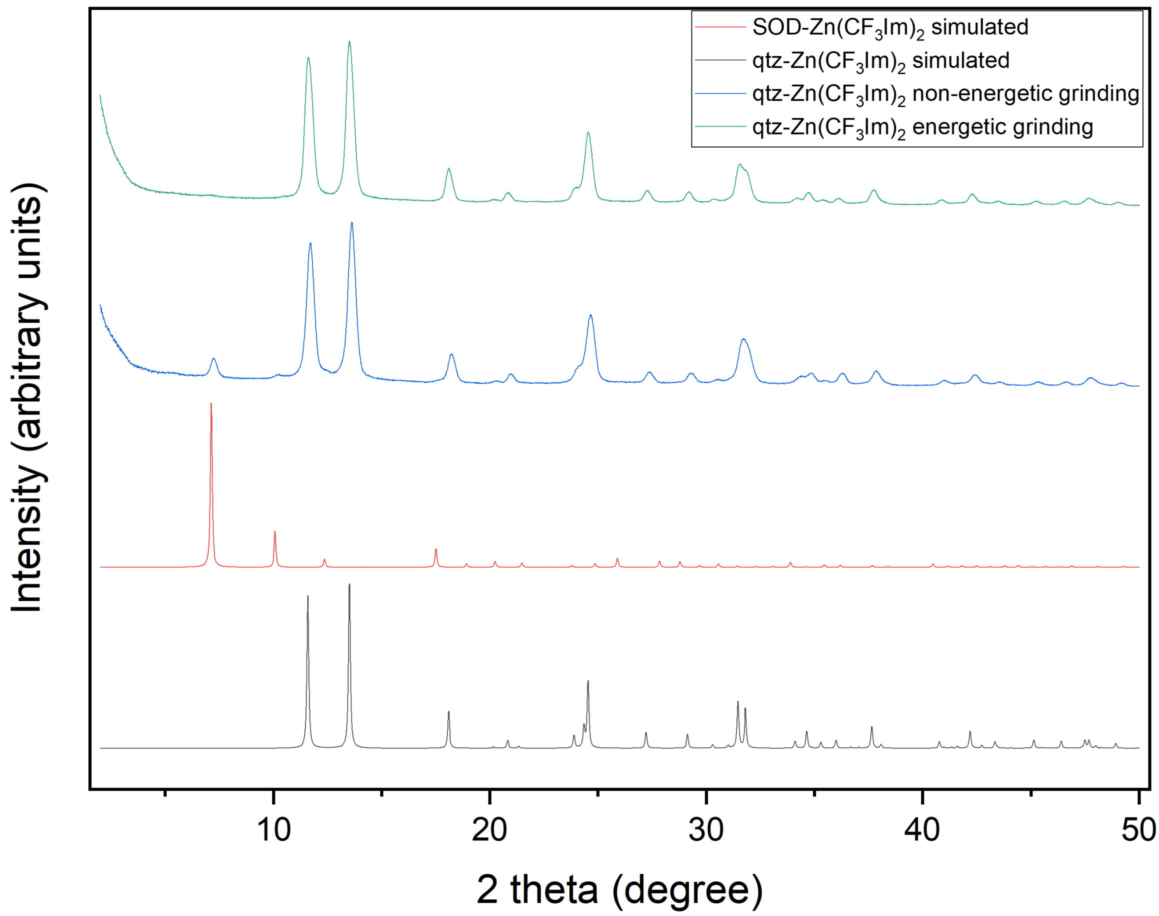

Figure S2. PXRD patterns of non-energetically ground qtz and energetically ground qtz mortar and pestle syntheses compared with the simulated patterns for SOD-Zn(CF_3Im)$_2$ and SOD-Zn(CF_3Im)$_2$.

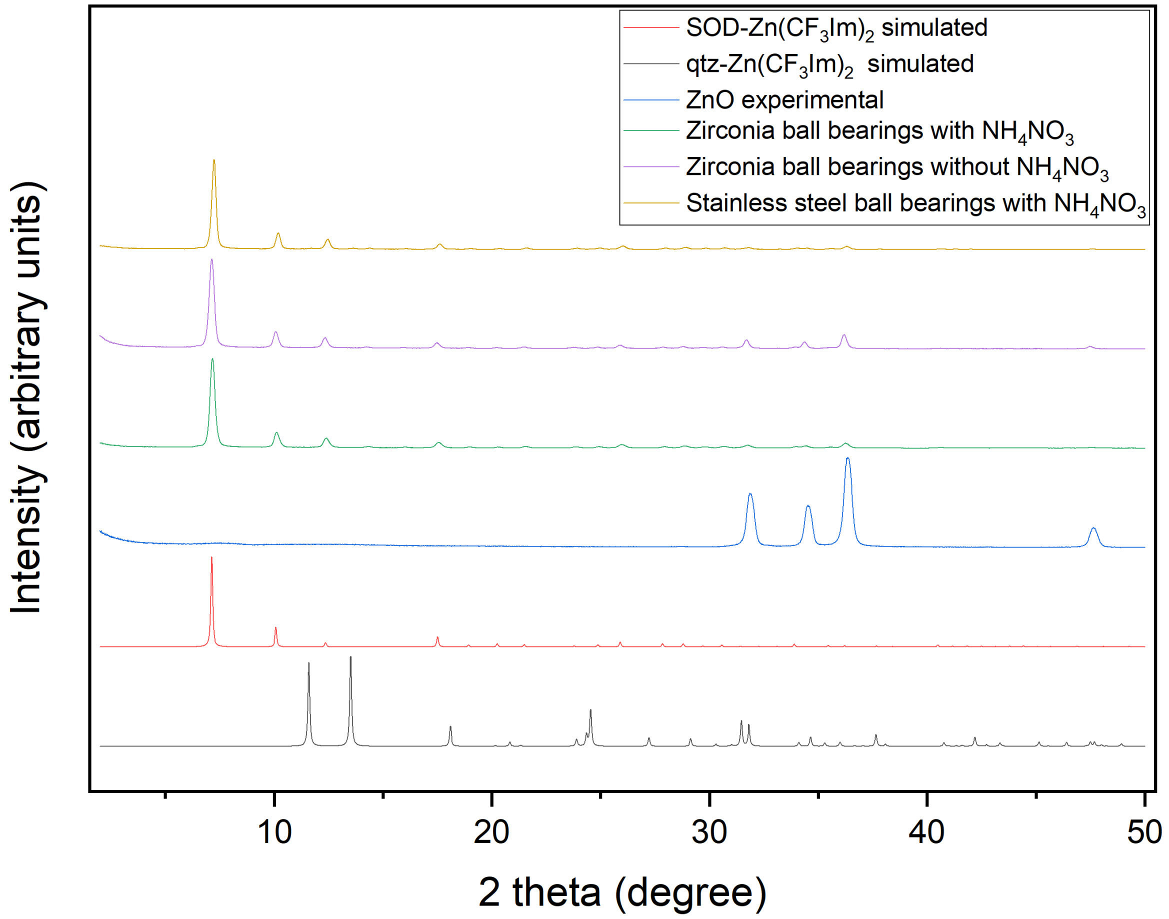

Figure S3. PXRD patterns of as-synthesised SOD-Zn(CF_3Im)$_2$ with stainless steel ball bearings and without catalytic salt NH_4NO_3.

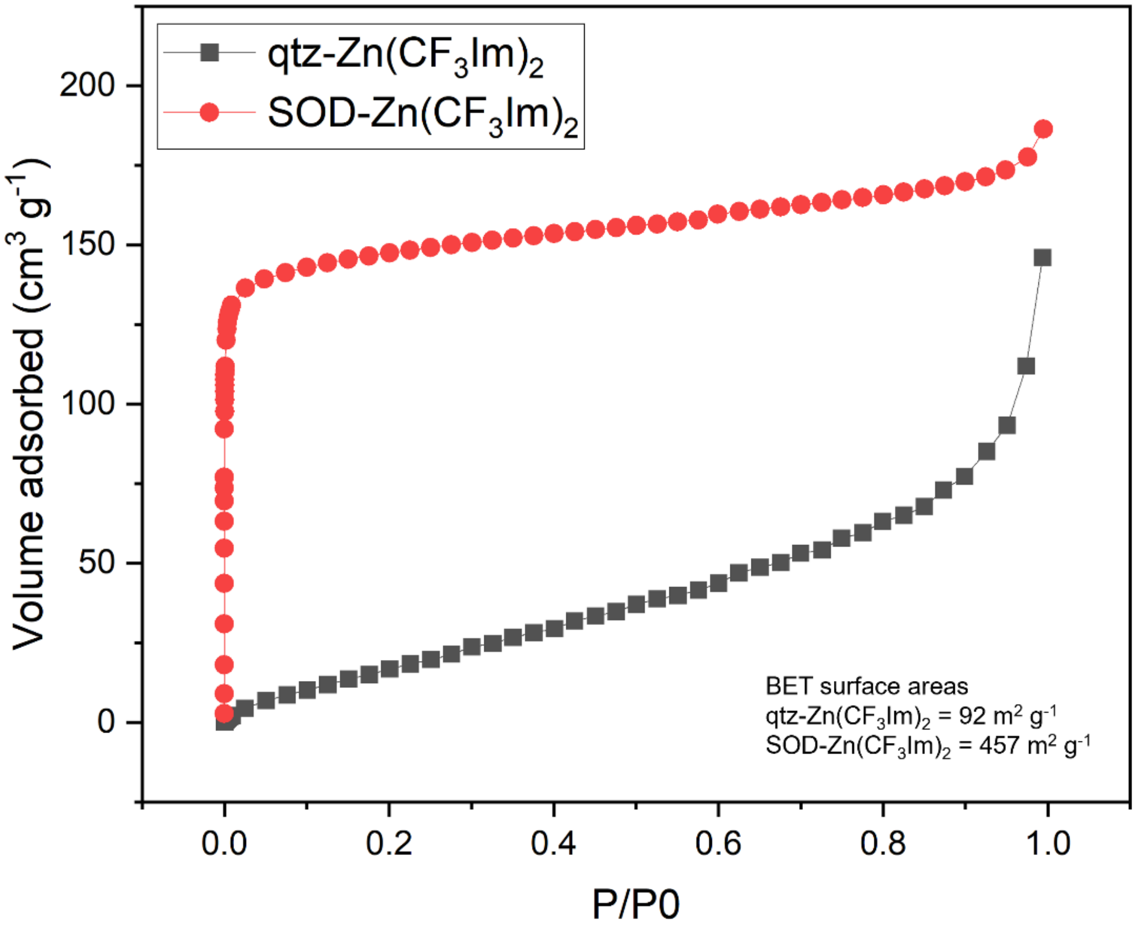

Figure S4. N_2 adsorption isotherms at 77 K of SOD-Zn(CF_3Im)$_2$ and qtz-Zn(CF_3Im)$_2$.

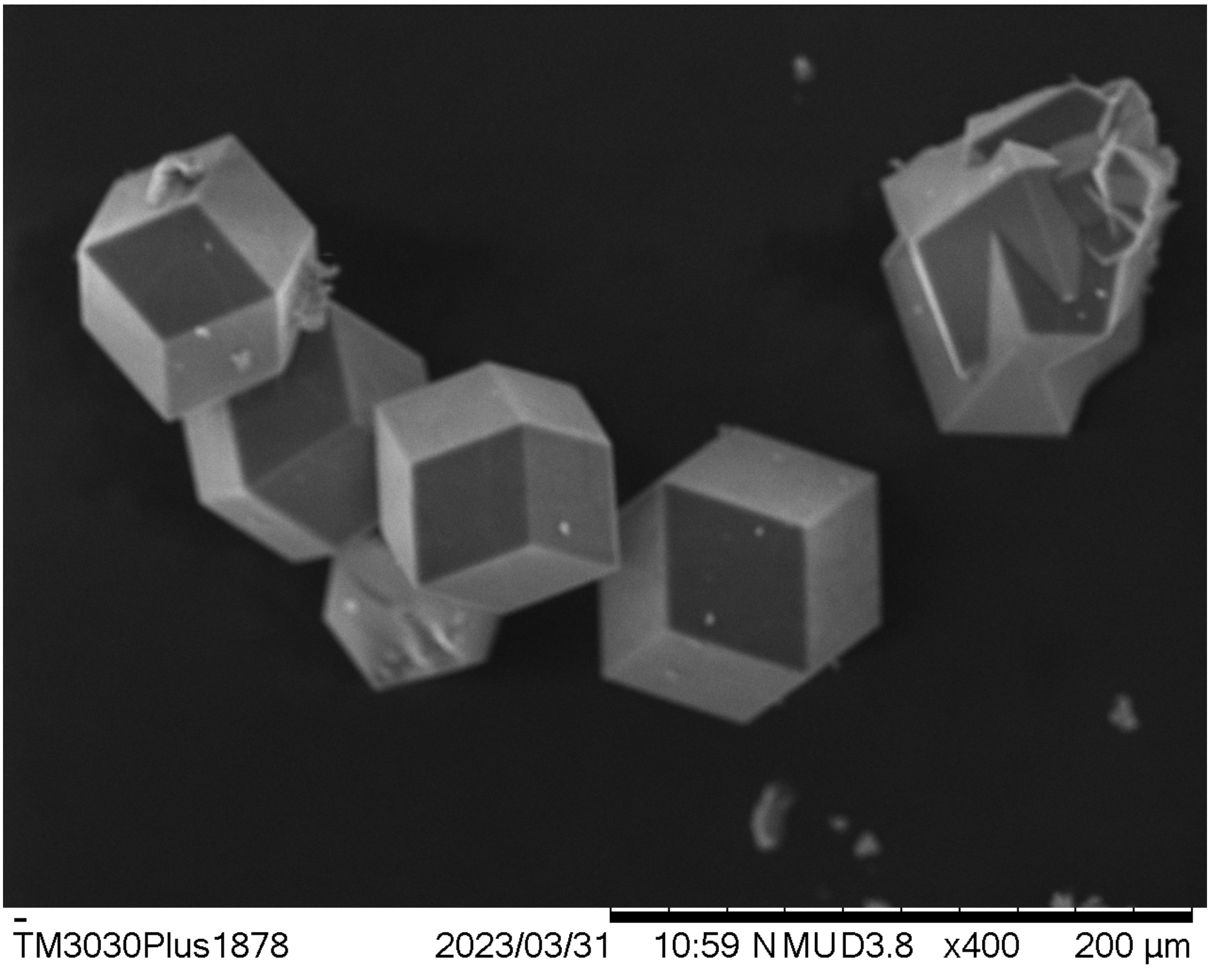

Figure S5. Scanning electron micrograph of as-synthesised crystals of ZIF-318.

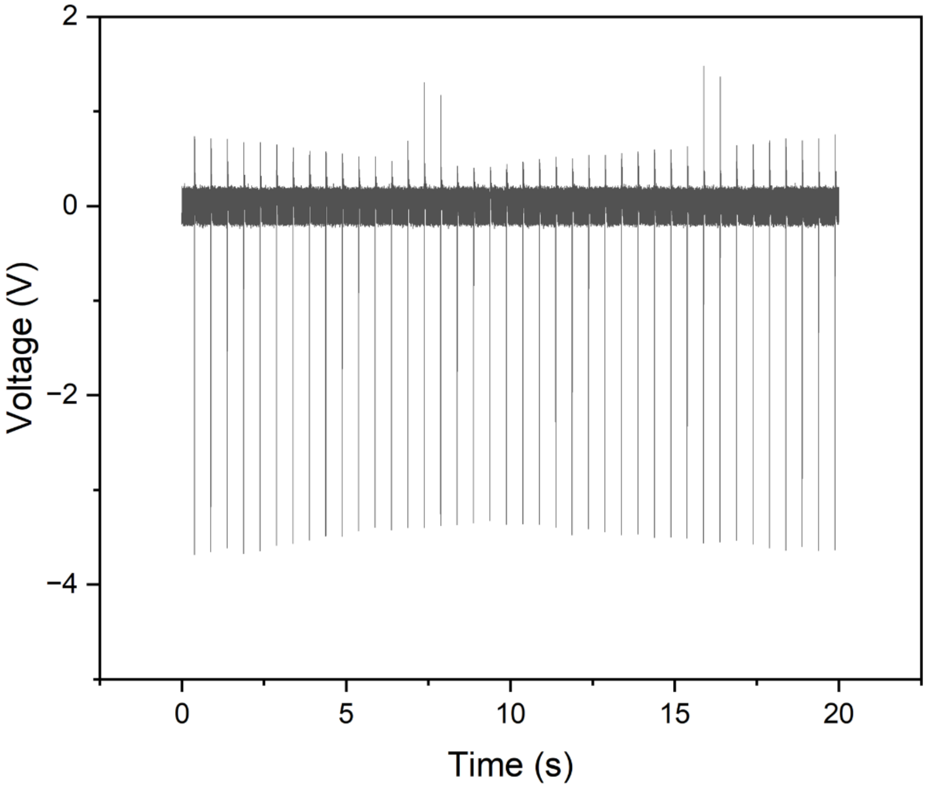

Figure S6. Pseudo voltage output recorded from electromagnetic shaker interference.

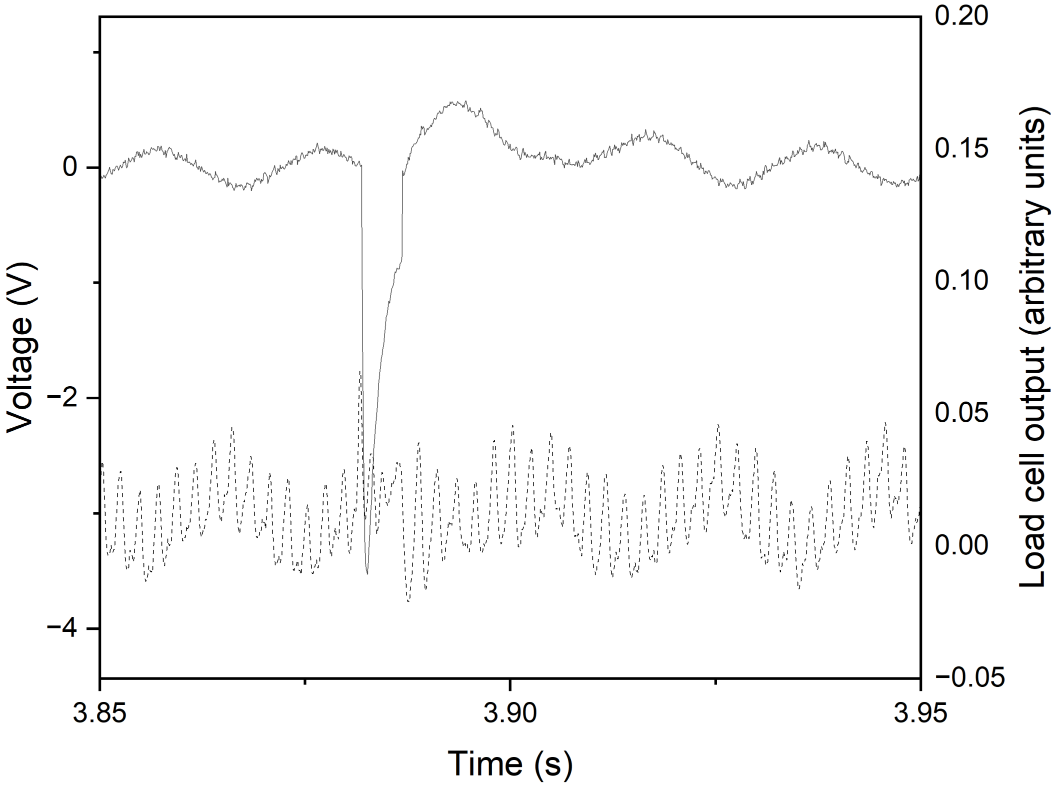

Figure S7. Peak shape of pseudo voltage output recorded from the electromagnetic shaker interference.

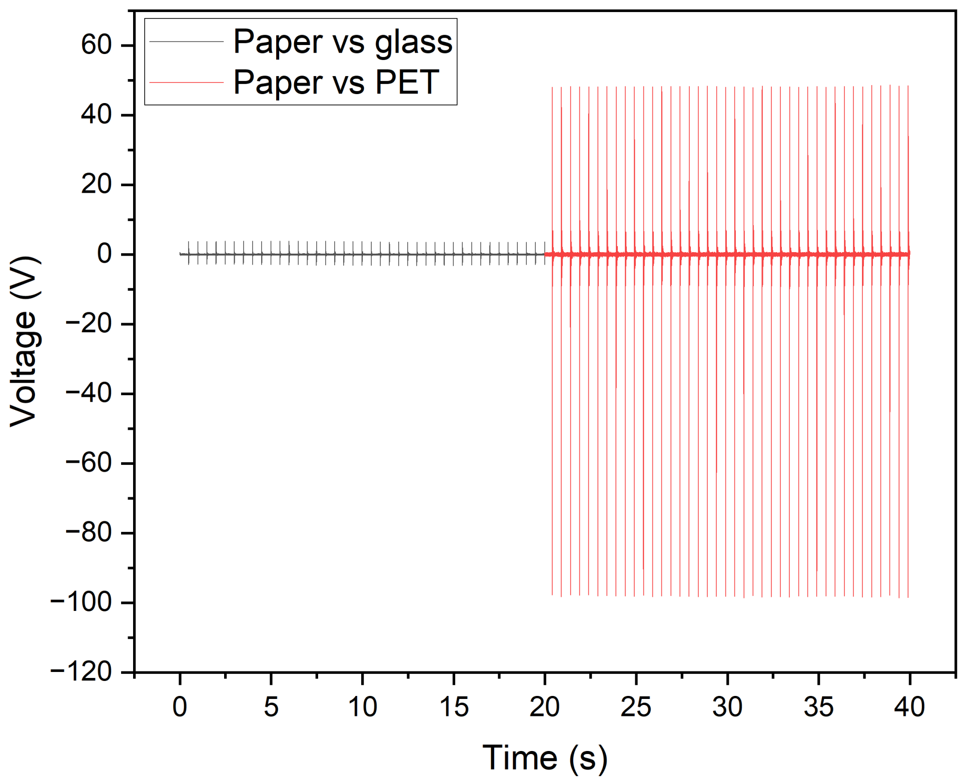

Figure S8. Triboelectric output of paper vs glass (black) and paper vs PET (red).

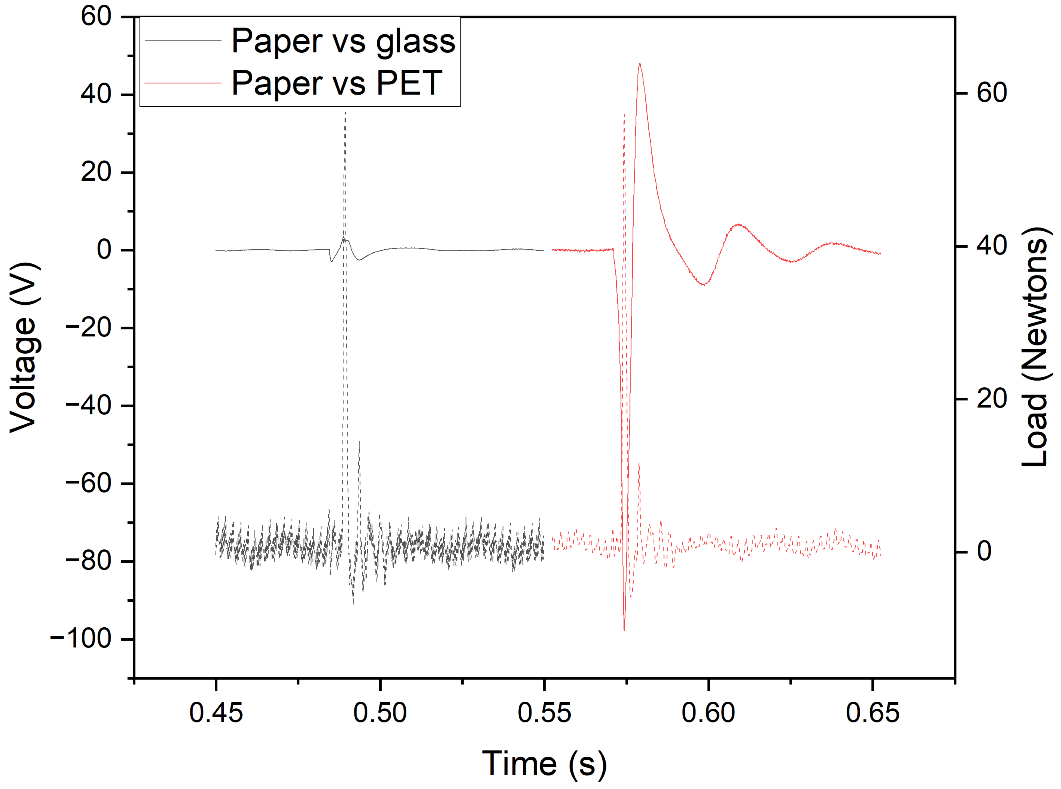

Figure S9. Peak shape of the single electrode voltage output of paper vs glass (black) and paper vs PET (red), the dashed line represents the force as detected by the load cell, plotted on the secondary y axis.

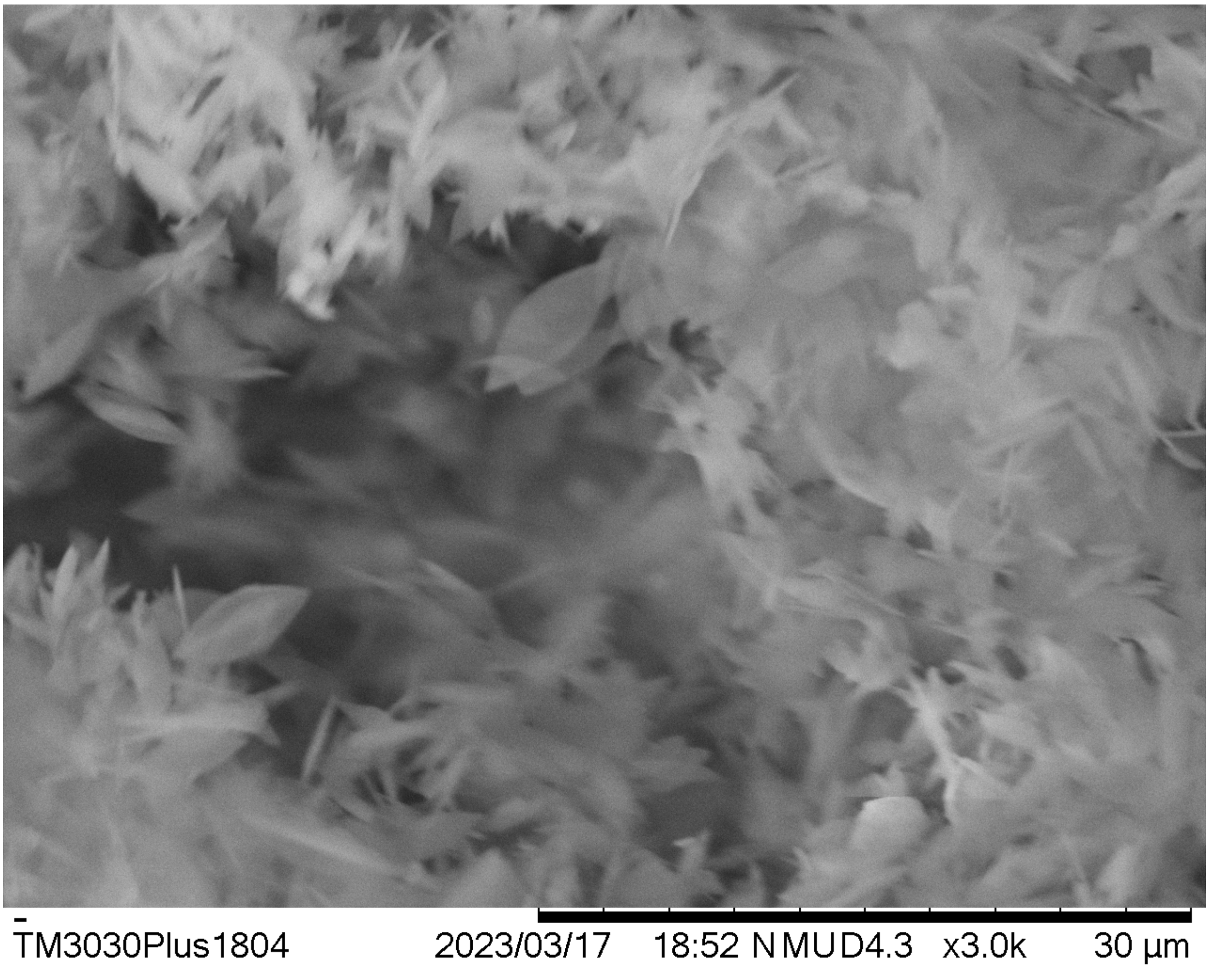

Figure S10. Scanning electron micrograph of as-synthesised ZIF-L particles.

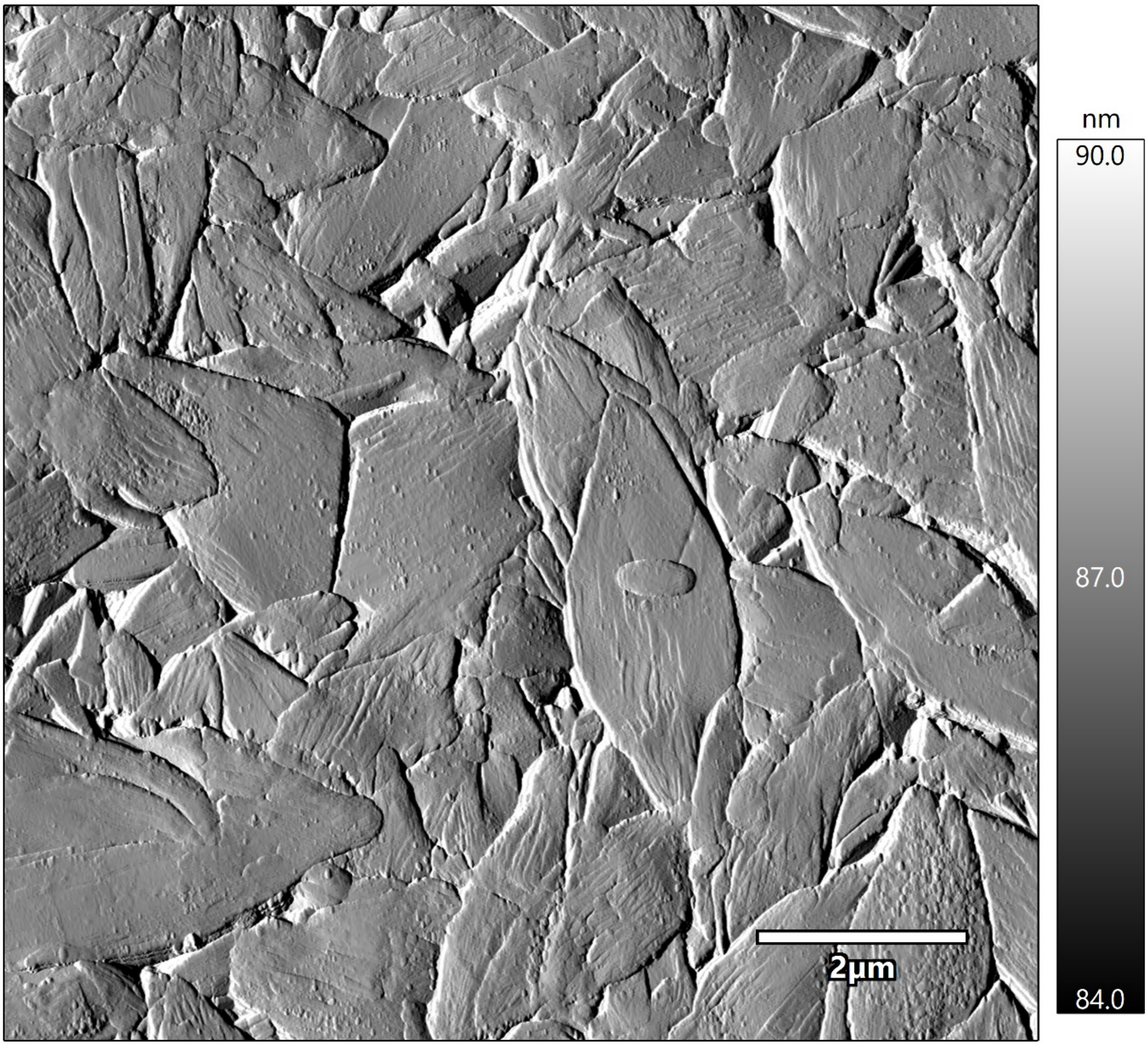

Figure S11. Amplitude-modulated mode atomic force micrograph of ZIF-L pellet showing oriented leaf particles.

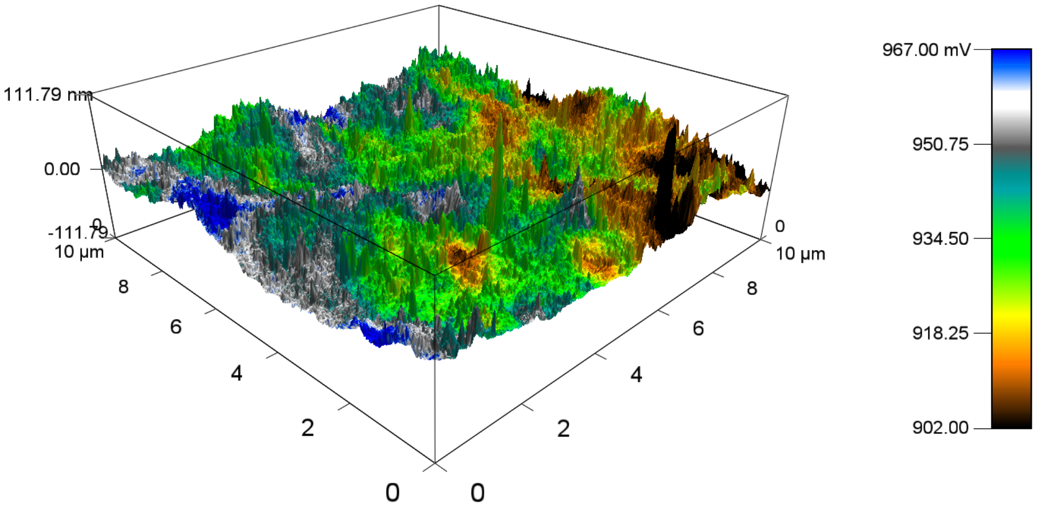

Figure S12. 3D map of the height profile topography with the KPFM surface potential overlaid as pseudocolour with corresponding voltage-colour scale of aluminium tape (foil side of tape).

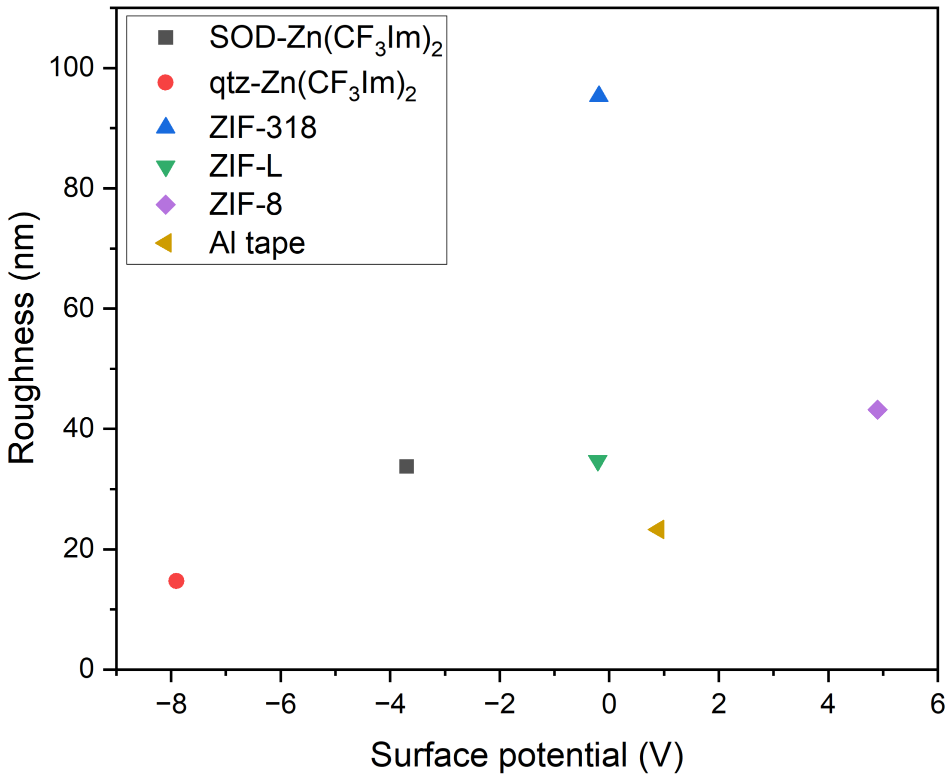

Figure S13. RMS surface roughness vs KPFM surface potential for the five ZIF samples and aluminium.

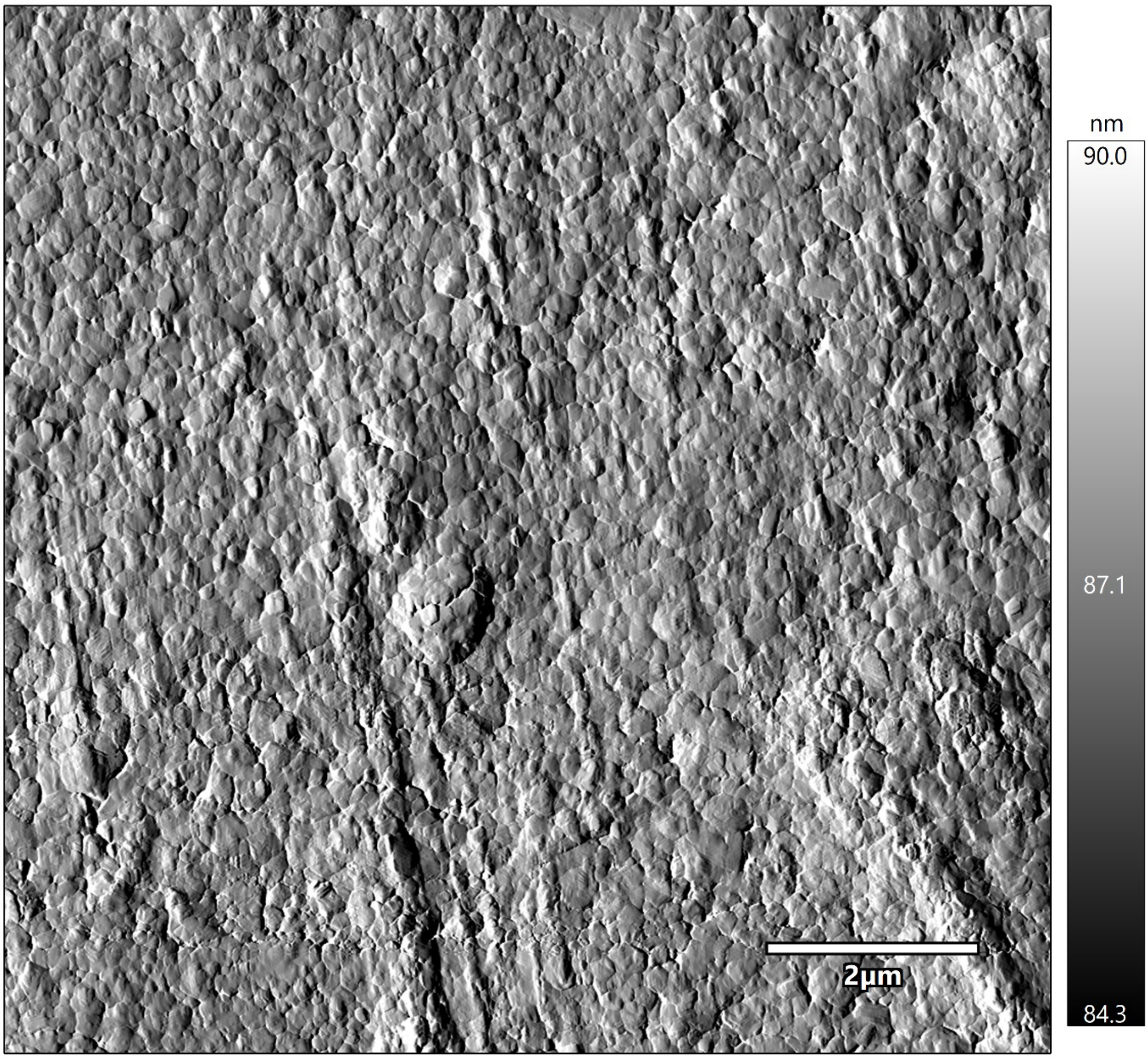

Figure S14. Amplitude-modulated mode atomic force micrograph of SOD-Zn(CF_3Im)$_2$ pellet.

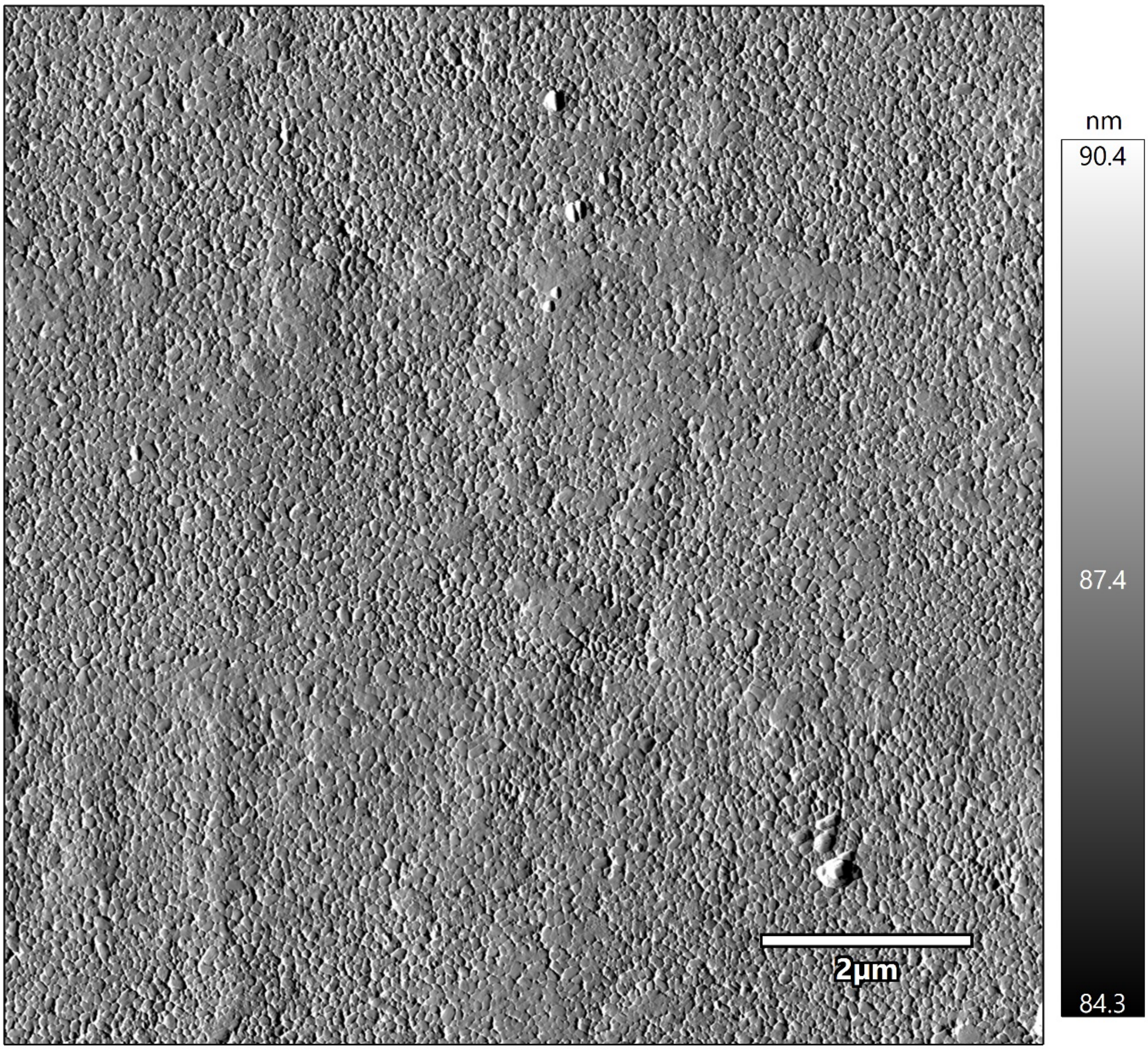

Figure S15. Amplitude-modulated mode atomic force micrograph of qtz-Zn(CF_3Im)$_2$ pellet.

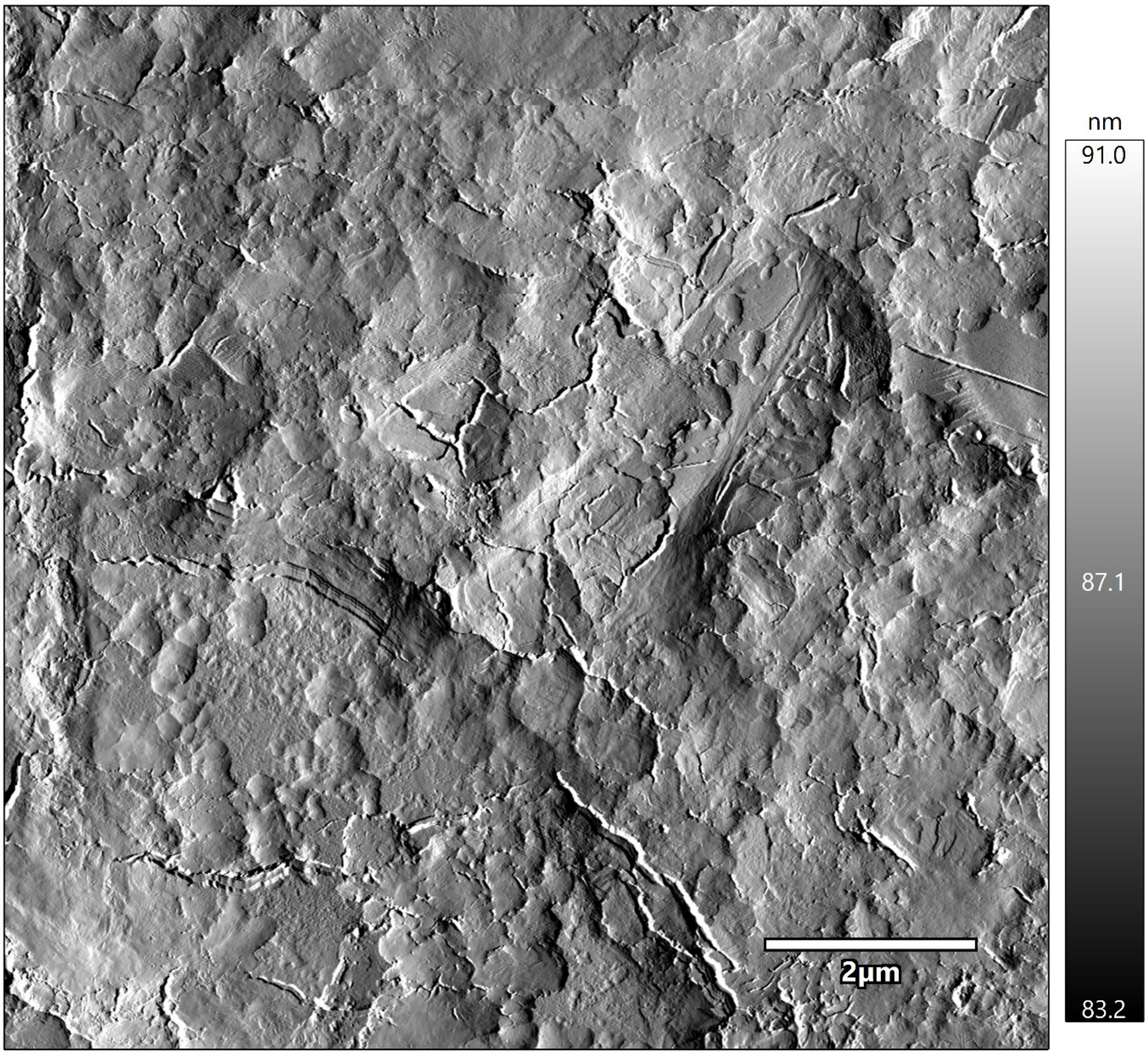

Figure S16. Amplitude-modulated mode atomic force micrograph of ZIF-318.

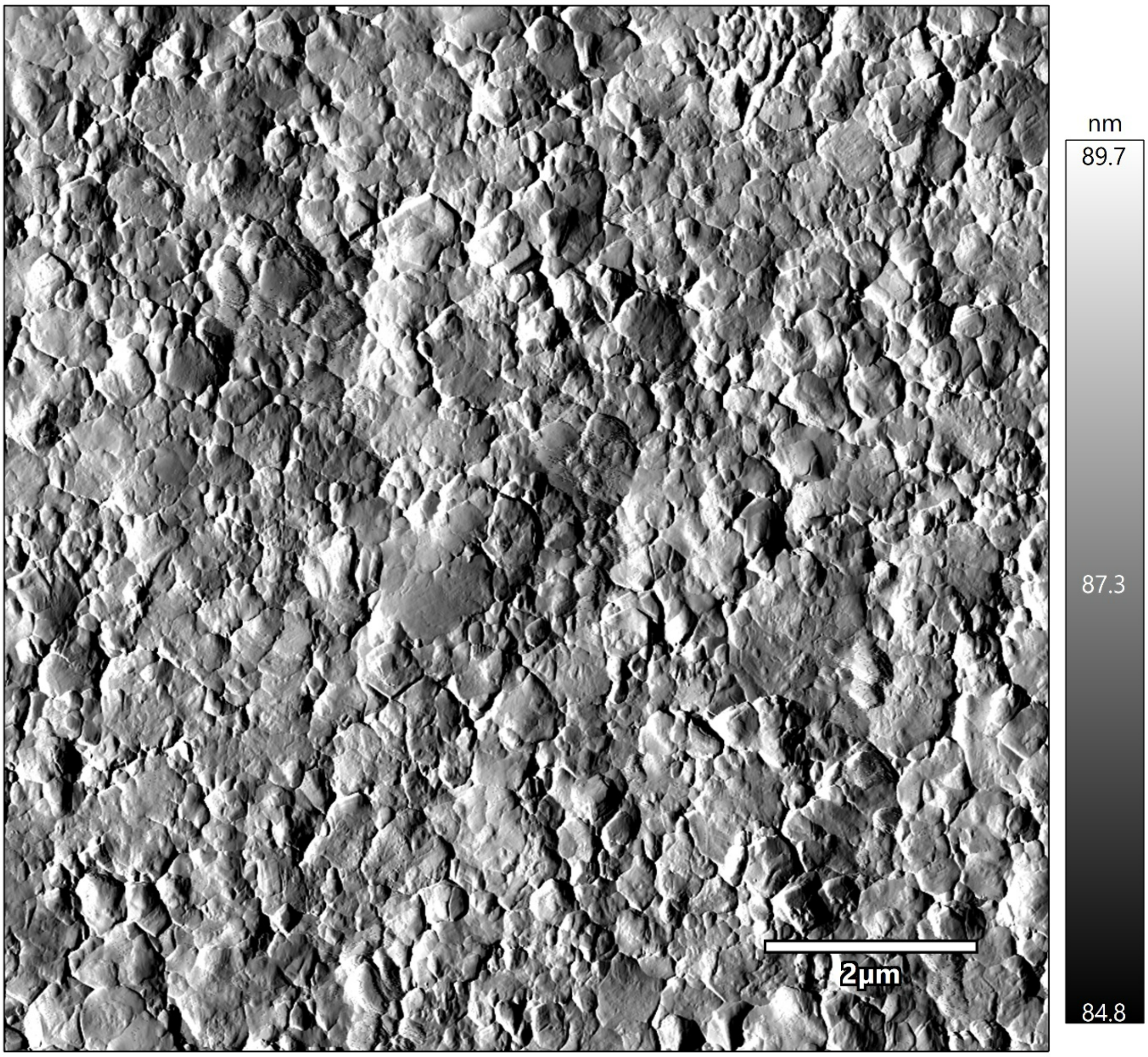

Figure S17. Amplitude-modulated mode atomic force micrograph of ZIF-8 pellet.

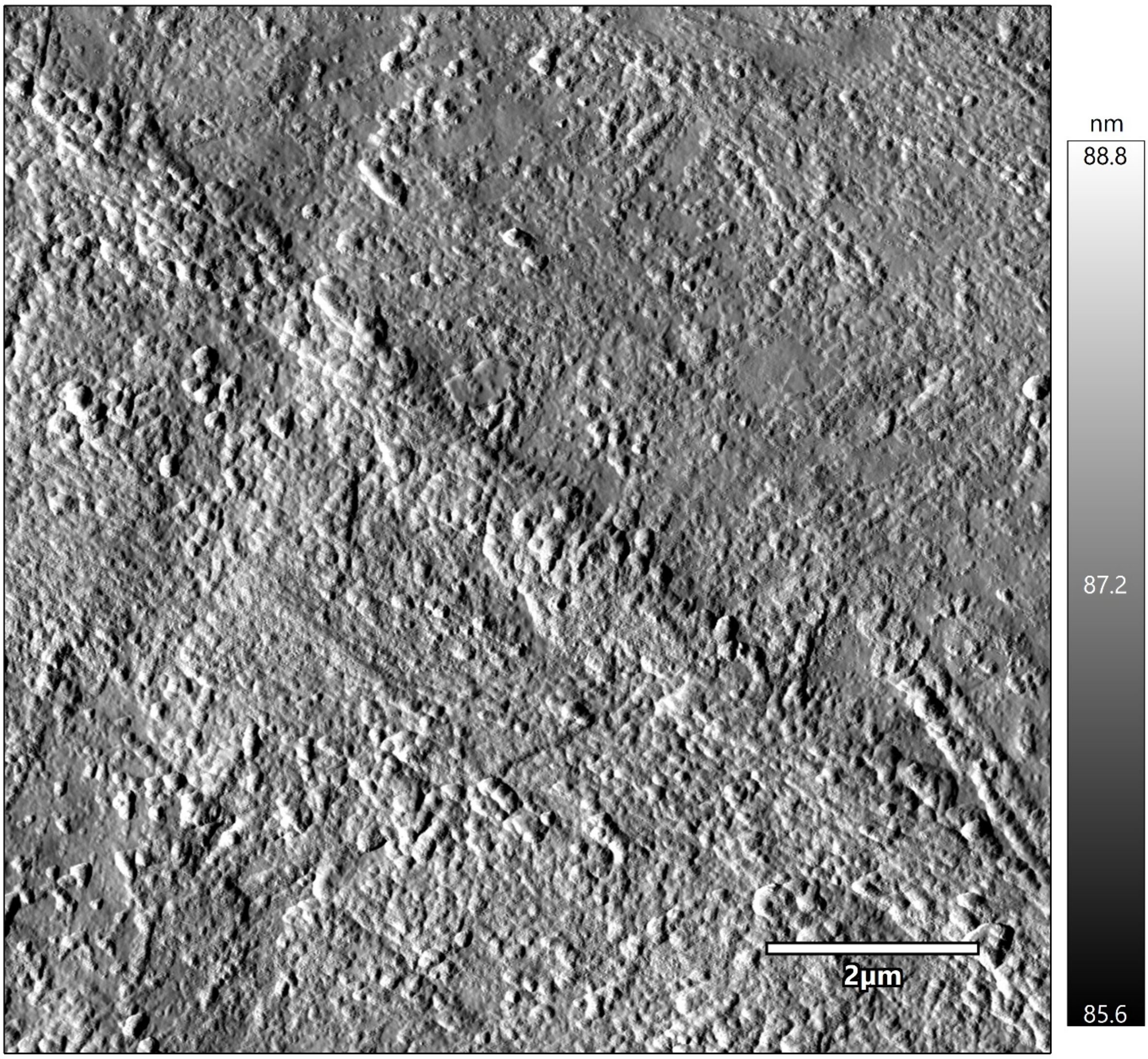

Figure S18. Amplitude-modulated mode atomic force micrograph of aluminium tape (metallic surface).

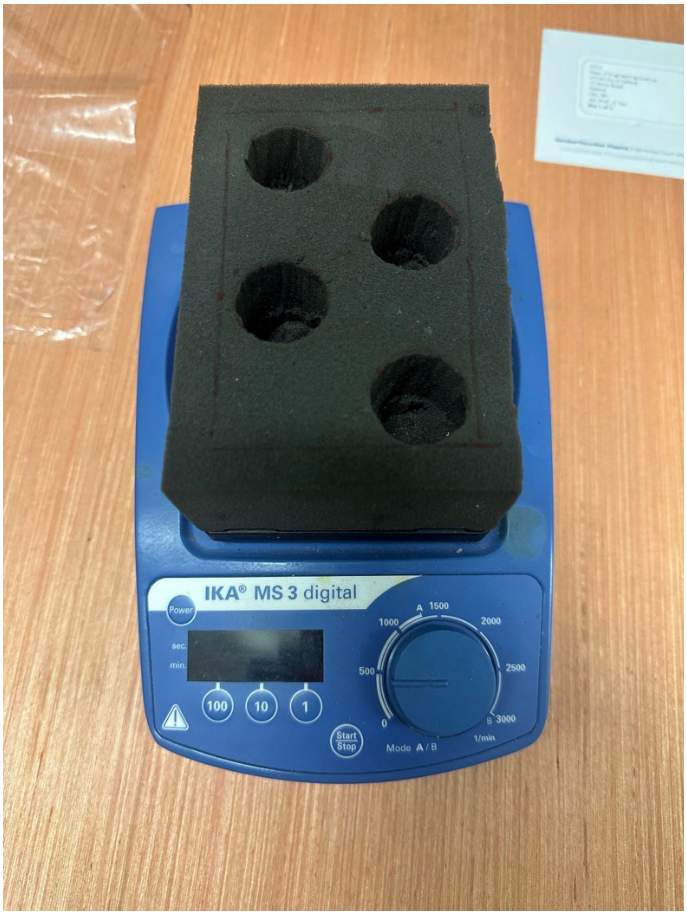

Figure S19. Vortex shaker with customised vial holder.

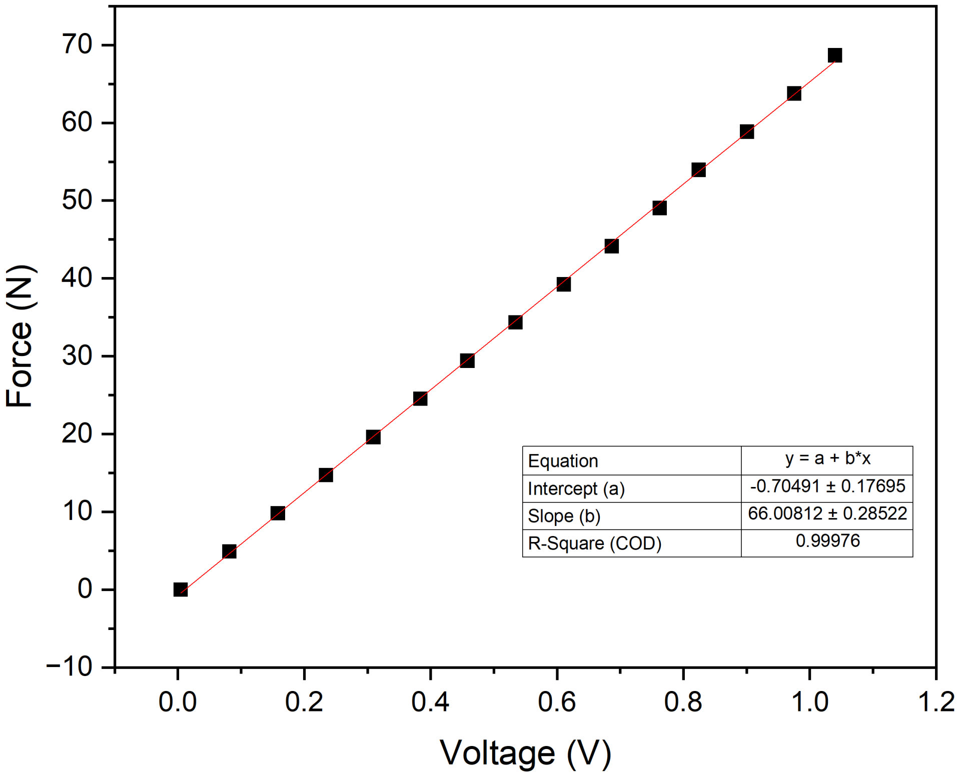

Figure S20. Load cell force calibration for voltage-to-force calibration.

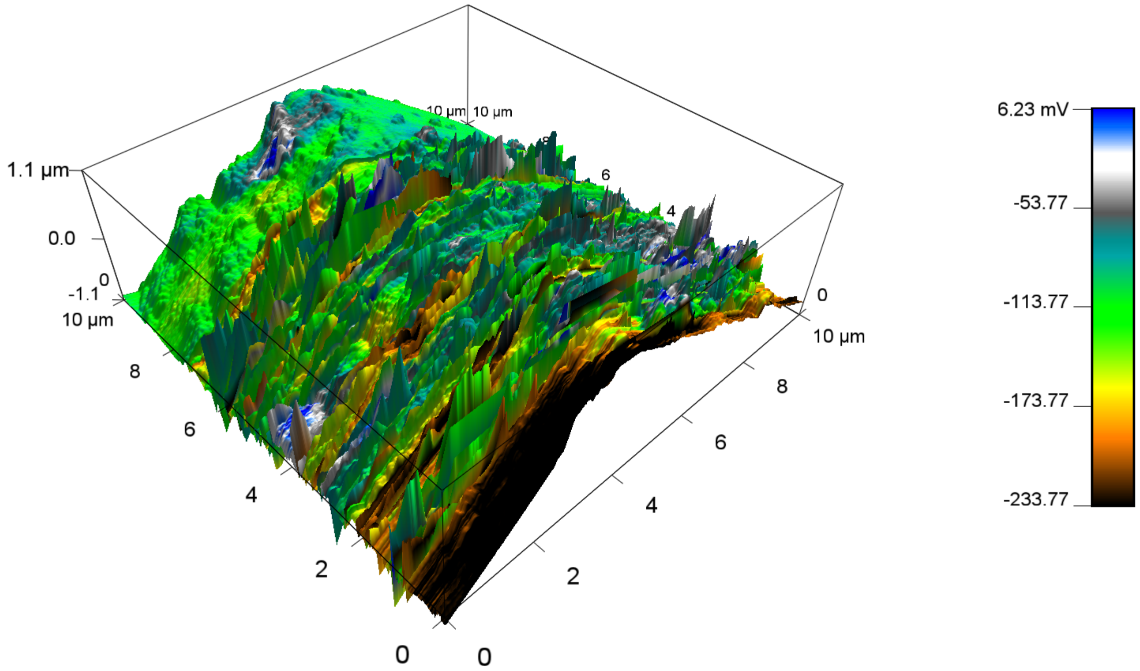

Figure S21. 3D map of the height profile topography with the KPFM surface potential overlaid as pseudocolour with corresponding voltage-colour scale of an edge of a ZIF-318 single crystal (scanning artefacts present in image).

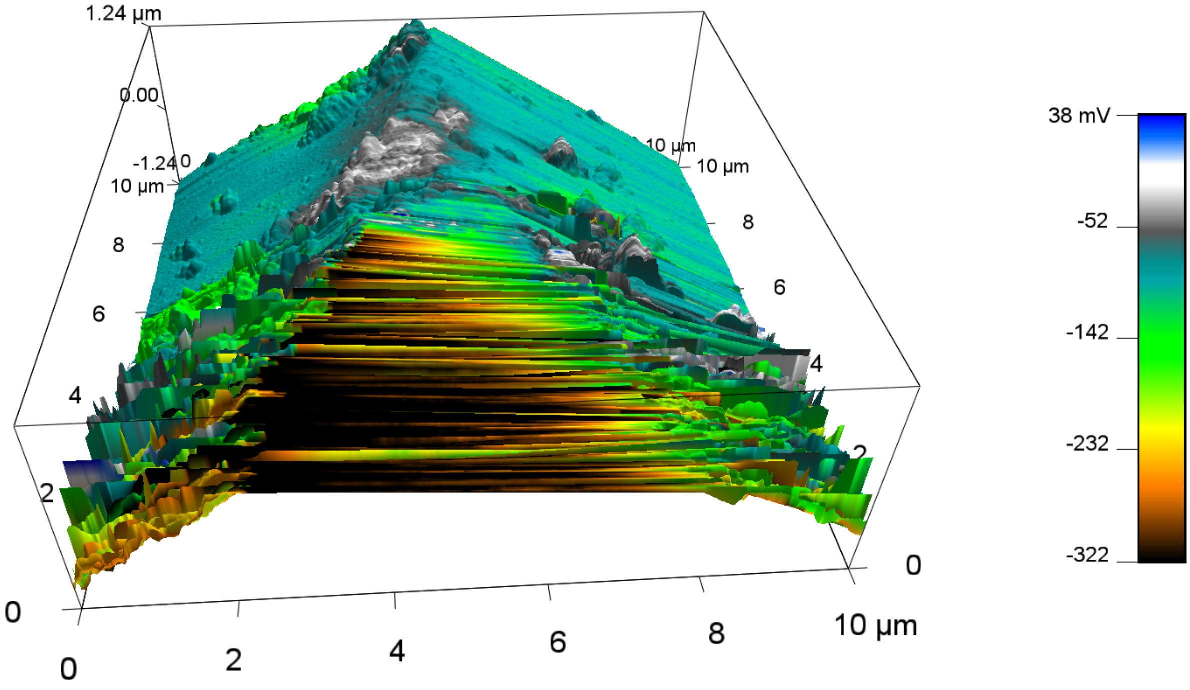

Figure S22. 3D map of the height profile topography with the KPFM surface potential overlaid as pseudocolour with corresponding voltage-colour scale of a ZIF-318 single crystal vertex (scanning artefacts present in lower part of image).

References

1. M. Arhangelskis, A. D. Katsenis, N. Novendra, Z. Akimbekov, D. Gandrath, J. M. Marrett, G. Ayoub, A. J. Morris, O. K. Farha, T. Friščić and A. Navrotsky, Theoretical Prediction and Experimental Evaluation of Topological Landscape and Thermodynamic Stability of a Fluorinated Zeolitic Imidazolate Framework, *Chem. Mater.*, 2019, **31**, 3777-3783.
2. S. S. Mondal, M. Hovestadt, S. Dey, C. Paula, S. Glomb, A. Kelling, U. Schilde, C. Janiak, M. Hartmann and H.-J. Holdt, Synthesis of a partially fluorinated ZIF-8 analog for ethane/ethene separation, *CrystEngComm*, 2017, **19**, 5882-5891.
3. Z.-X. Low, A. Razmjou, K. Wang, S. Gray, M. Duke and H. Wang, Effect of addition of two-dimensional ZIF-L nanoflakes on the properties of polyethersulfone ultrafiltration membrane, *J. Membr. Sci.*, 2014, **460**, 9-17.
